# Causal associations of anthropometric measurements with osteoarthritis: A Mendelian randomization study

**DOI:** 10.1371/journal.pone.0279198

**Published:** 2023-01-30

**Authors:** Yang Sun, Yue Li, Tiecheng Yu, Jiting Zhang

**Affiliations:** 1 Department of Orthopedics, The First Hospital of Jilin University, Jilin Changchun, China; 2 Department of Social Psychiatry, The Affiliated Brain Hospital of Guangzhou Medical University, Guangzhou, Guangdong, China; Yale University School of Medicine, UNITED STATES

## Abstract

**Objective:**

We believe that there is a causal relationship between waist circumference and knee osteoarthritis. To confirm the hypothesis, we have conducted this study.

**Methods:**

Genetic variants associated with the five anthropometric variables were obtained from previous large-scale genomewide association studies. Summary-level data on osteoarthritis were obtained from the UK Biobank. The univariable and multivariable MR framework were used to evaluate the associations. The two-sided p value was considered to be statistically significant at 0.01 (where p = 0.05/5) after Bonferroni correction for the five exposure variables.

**Results:**

In the univariable MR, there was evidence of a detrimental effect of height, weight, BMI, waist circumference, and hip circumference on osteoarthritis risk in the main IVW analyses (height: OR 1.115, 95% CI 1.054–1.180; weight: OR 1.765, 95% CI 1.650–1.889; BMI: OR 1.952, 95%CI 1.841–2.068; waist circumference: OR 2.140, 95% CI 1.994–2.296; hip circumference: OR 1.719, 95% CI 1.600–1.846). And the analyses on knee osteoarthritis and hip osteoarthritis yielded similar results. However, the multivariable MR showed that only waist circumference was causally associated with osteoarthritis, after adjusting for the confounding exposure effects (waist circumference: OR 1.877, 95% CI 1.286–2.739). Such association was also repeated in the analyses on knee osteoarthritis but not hip osteoarthritis.

**Conclusion:**

Our study highlighted the causal associations between waist circumference and knee osteoarthritis risk.

## Introduction

Osteoarthritis (OA) is a musculoskeletal disorder that causes degradation of synovial joints. It is the most common joint disease, and most frequently affects the hip, knee, spine, and the small joints of the hand and the foot. OA causes loss of articular cartilage, formation of osteophytes, changes in subchondral bone, and synovitis [1]. It is a complex disease that affects the whole joint, with multiple biochemical, genetic, biological, morphological, biomechanical, and environmental factors contributing to disease occurrence [1, 2]. The heritable contribution to primary OA susceptibility has been established by twin and sibling studies to be about 50% (range 40–65%) [3]. So far, candidate gene studies and genome-wide association scans have established 18 OA-associated loci. These findings account for 11% of the heritability, explaining a rather small fraction of the genetic component. Osteoarthritis (OA) is the most common type of arthritis and a major cause of disability among adults [4]. It contributes to 2.4% of all years lived with disability (YLD) globally and ranks as the leading contributor [5]. Hip and knee OA is estimated to affect 5% of the world’s population and will continue to rise [5].

It is well known that obesity is an important predisposing factor for OA [6]. Therefore, anthropometric measurements reflecting the degree of obesity are also closely associated with OA risk [7, 8]. Heritability studies provide evidence for a substantial genetic contribution of increased anthropometric measurements to OA, with heritability estimates ranging from 0.6 to 0.8 [9–11]. Some genome-wide association studies found genetic overlap between high BMI and osteoarthritis [12]. Unfortunately, this association has not been adequately illuminated. Firstly, there are various types of obesity [13]. Pathological obesity, such as central (visceral) obesity, is more damaging to health than general obesity [14, 15]. However, the degree of reflection on different types of obesity is not consistent across different anthropometric measurements. For example, waist circumference is much more sensitive to central (visceral) obesity than body mass index (BMI) [16]. An observational study has demonstrated that individuals at OA risk with normal weight but central obesity have greater declines in physical functioning and physical activity based on reliable instruments than those with normal BMI and waist circumference [17]. The results suggested the necessity of assessing waist circumference in individuals with normal BMI to further stratify their risk and highlighted the additive effect of central adiposity in individuals at OA risk. Therefore, we believe that there are differences in the effect of different anthropometric measurements on the risk of OA and this may indirectly reflect differences in the effect of obesity type. Secondly, assessing causal effects in observational studies is challenging due to environmental confounders or reverse causation though several prior studies have examined the relationship between anthropometric measurements and OA [18–20]. Thirdly, MR results involving anthropometric and OA risks are inconsistent. For example, Funck-Brentano et al. have indicated that BMI exerts a major causal effect on the risk of OA [21]; however, such positive association was not replicated in Hartley’s study [22]. This discrepancy may be attributable to the incompleteness of exposure and the limitation of univariable MR.

Mendelian randomization (MR) [23] has proven to be a reliable method for overcoming the limitations of observational studies and assessing causality. As genetic variants are randomly assigned at conception, their association with the outcome is less influenced by external confounders.

The aim of our study was to use the MR framework to assess the effects of anthropometric measurements on risk of OA. Given that different measurements are correlated and pleiotropic, we further leveraged multivariable MR (MVMR) methods developed in 2015 [24] to adjust for potential confounders. The assessment of direct causal effects of each anthropometric measurement to OA can provide unbiased genetic causation between exposure and outcome. Additionally, evaluation and illumination of the association between anthropometric measurements and OA may be helpful to public health and clinical prevention.

## Methods

### Overall study design

In this study, we have extracted instrumental variables (IVs) for anthropometric measurements (including height, weight, BMI, waist circumference, and hip circumference) from publicly available summary statistics, and assessed the single causal effects on the OA, knee OA, and hip OA risk by applying univariable MR analyses. Secondly, we performed multivariable MR analyses to estimate the direct effects of each anthropometric measurement on the OA, knee OA, and hip OA, after adjustment for the confounding exposure effects.

### Data source

The summary statistics data on OA, knee OA, and hip OA were retrieved from the European Bioinformatics Institute, which included 77,052 cases and 378,169 controls with acceptable imputation quality [25].

Summary statistics for anthropometric measurements were all acquired from the UK Biobank. The genetic variants were reclaimed from the largest GWAS of height (N = 461,950) [26], weight (N = 461,632) [27], BMI (N = 461,460) [28], waist circumference (N = 462,166) [29], and hip circumference (N = 462,117) [30]. The details of the instruments used for exposures are presented in **Table 1**.

**Table 1:** Details of the instruments used for exposures.

Exposure	Consortium	Sample size	Population	Outcome	No.SNPs	F-statistics	Pleiotropy		Heterogeneity (MR-Egger)	
							**Intercept**	**P value**	**Q**	**P value**
Height	UK Biobank	461,950	European	Osteoarthritis	748	52.773	1.608E-04	0.882	1928.636	1.494E-105
				Knee osteoarthritis	751	52.701	3.000E-04	0.806	1652.124	6.078E-70
				Hip osteoarthritis	751	52.701	-4.417E-04	0.780	1751.316	4.813E-82
Weight	UK Biobank	461,632	European	Osteoarthritis	486	27.347	1.739E-03	0.239	1027.147	3.129E-41
				Knee osteoarthritis	484	27.289	2.473E-03	0.092	1020.106	9.547E-41
				Hip osteoarthritis	483	27.357	-1.793E-03	0.396	883.739	3.665E-26
BMI	UK Biobank	461,460	European	Osteoarthritis	440	24.870	1.442E-03	0.322	725.923	1.367E-16
				Knee osteoarthritis	441	25.266	2.307E-03	0.455	745.058	3.509E-18
				Hip osteoarthritis	438	24.819	-3.998E-03	0.058	638.286	8.161E-10
Waist circumference	UK Biobank	462,166	European	Osteoarthritis	362	22.914	8.898E-04	0.576	542.029	1.654E-09
				Knee osteoarthritis	362	22.914	1.552E-03	0.438	572.499	6.501E-12
				Hip osteoarthritis	361	22.861	-5.980E-04	0.800	502.761	7.754E-07
Hip circumference	UK Biobank	462,117	European	Osteoarthritis	404	24.825	4.501E-04	0.807	957.917	2.440E-47
				Knee osteoarthritis	404	24.825	2.928E-03	0.181	907.603	4.404E-41
				Hip osteoarthritis	400	24.796	-3.731E-03	0.166	858.625	6.473E-36

No.SNPs: number of single nucleotide polymorphism; BMI: body mass index

We also could download GWAS summary statistics of IBD and BMD from the publicly available GWAS catalog website (https://www.ebi.ac.uk/gwas/downloads/summary-statistics) or IEU GWAS database (https://gwas.mrcieu.ac.uk/datasets/).

### Statistical analysis

We conducted fifteen separate two-sample MR analyses to test the potential causal associations between anthropometric measurements and OA. The three key assumptions [23, 31] underlying the two-sample MR approach are:

- The genetic variants must be strongly associated with the exposure;
- The variants must affect the outcome only through their effect on exposure;
- The variants must be independent of any confounders of the association between the exposure and the outcome.

TwoSample MR R packages were used for all analyses. All genetic variants reaching genome-wide significance (p<5×10e-8) and being independent (10000 kb pairs apart and R2≤0.001) were selected as instruments for the MR analysis. The exposure and outcome GWAS provided SNP effects and corresponding standard errors [32]. We filtered out palindromic SNPs with intermediate allele frequencies after harmonizing exposure and outcome data [33]. We used the PhenoScanner V2 database to detect potential pleiotropy among the SNPs included in this study [34]. Genetic variants that were found to be related to potential confounding factors were excluded from the analyses. And F statistic was calculated to assess the strength of the selected SNPs.

For MR methods, the inverse variance–weighted (IVW) method was identified as the primary MR analysis. Weighted median and MR-Egger-based regression methods were incorporated to ensure the conclusions were reliable since the IVW method provides consistent estimates only when all genetic variants are valid IVs [23, 32, 35]. When 100% genetic variants are invalid IVs, the MR-Egger regression provides reliable estimates; in contrast, the weighted median requires that 50% of the weight come from valid IVs. In terms of efficiency, however, weighted median estimates are typically almost as accurate as IVW estimates; both are significantly more accurate than MR-Egger estimates, with MR-Egger regression estimates being especially inaccurate when all IVs are associated with exposure to similar magnitudes [36]. To assess potential IV pleiotropy, we also conducted the MR pleiotropy residual sum and outlier (MR-PRESSO) test [37], MR-Egger intercept test [38], and Cochran Q heterogeneity test [39]. The "leave-one-out" sensitivity analyses were also performed to detect potentially influential SNPs [40].

MRlap is an approach to perform two-sample Mendelian Randomization (MR) analyses using (potentially) overlapping samples, relying only on GWAS summary statistics. MR estimates can be subject to different types of biases due to the overlap between the exposure and outcome samples, the use of weak instruments and winner’s curse. Our approach simultaneously accounts and corrects for all these biases, using cross-trait LD-score regression (LDSC) to approximate the overlap. Estimating the corrected effect using our approach can be performed as a sensitivity analysis: if the corrected effect does not significantly differ from the observed effect, then IVW-MR estimate can be safely used. However, when there is a significant difference, corrected effects should be preferred as they should be less biased, independently of the sample overlap. The package builds up on the GenomicSEM R-package to perform cross-trait LDSC and the TwoSampleMR R-package for inverse-variance weighted (IVW-)MR analysis (and instruments pruning).

**Fig 1:**
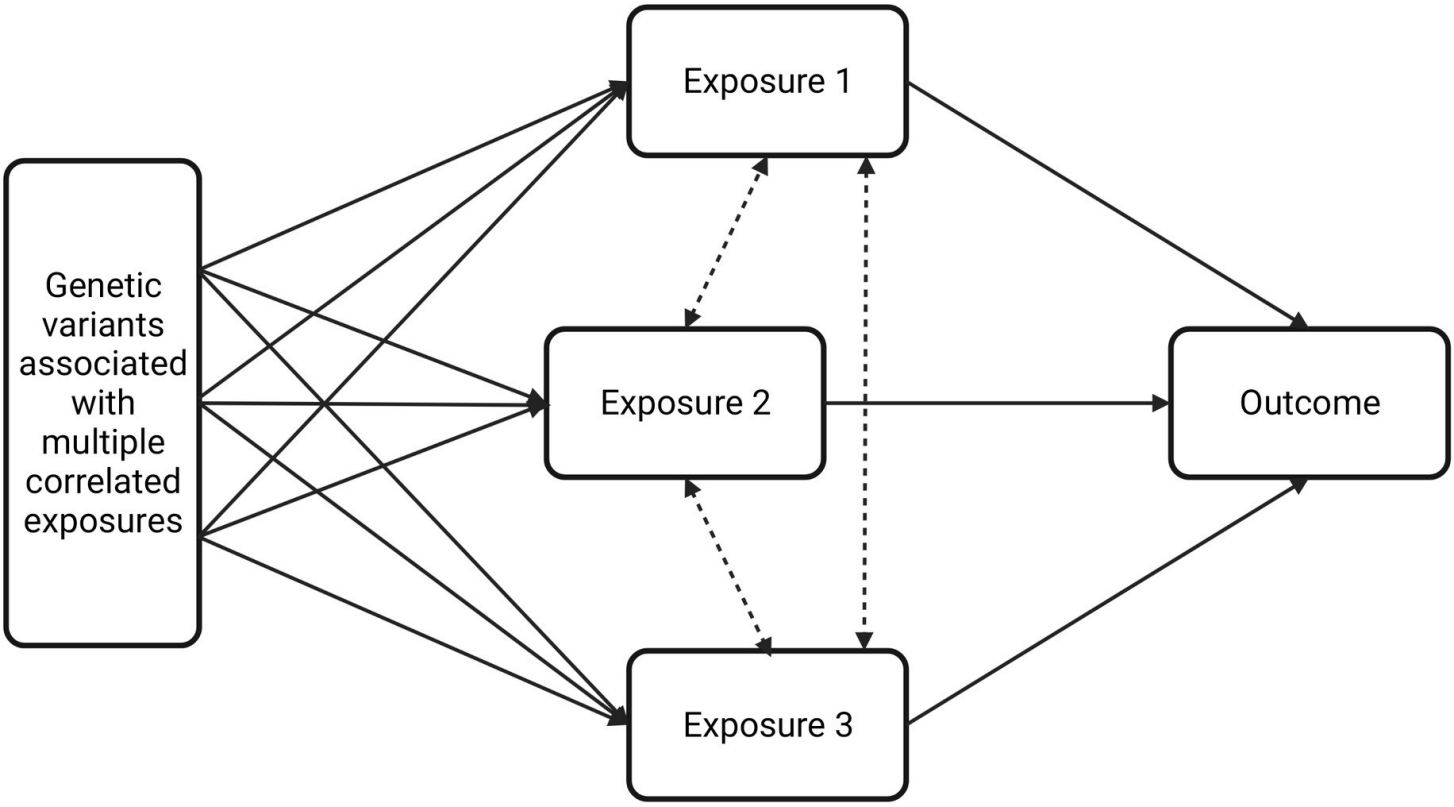
Schematic representation of multivariable Mendelian randomization analysis.

Multivariable MR (MVMR) is a novel extension to MR that incorporates genetic variants associated with multiple, potentially correlated exposures to calculate the effect of each exposure on a single outcome [41]. MVMR used the same IVs from univariable MR analysis. For this approach, the genetic variants do not have to be exclusively linked to a single exposure, but with a set of measured exposures, although it still needs to meet equivalent instrumental-variable assumptions [42]. The method gives a direct causal estimation for each exposure, taking into account the association between that exposure and the IVs with the other exposures in the analysis **(Fig 1)**. The anthropometric variables used in the MVMR analysis inclued: height, weight, BMI, waist circumference, and hip circumference.

### Ethical approval

All studies included were permitted by their academic ethics review committees, and each participant signed written informed consent. Since no primary data were used in this study, ethical approval was not required.

## Results

All genetic association estimates used in the univariable and MVMR analyses are presented in **S1-S18 Tables in S2 File** and visualized in **S1-S15 Figs in S1 File**. The intercept of the MR-Egger regression indicated that there was no directional pleiotropy among the SNPs associated with exposures **(Table 1)**. The F-statistics of instrumental variables ranged between 22.861 and 52.773, all >10, indicating no evidence of weak instrument bias **(Table 1)**.

### The causal effect of anthropometric measurements on OA

**Fig 2:**
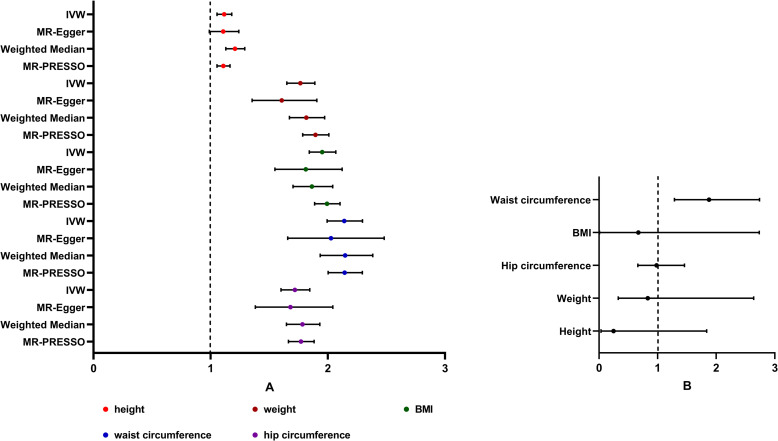
Forest plot for univariable and multivariable MR analyses of the causal effect of anthropometric measurements on osteoarthritis. A. Estimated causal effects of anthropometric measurements on osteoarthritis using univariable MR. The genetically predicted height, weight, BMI, waist circumference, and hip circumference are included. Inverse-variance weighted (IVW), MR-Egger, weighted median and MR-PRESSO represent different Mendelian randomization models. B. Estimated causal effects of anthropometric measurements on osteoarthritis using multivariable MR. MVMR results are from adjusting all 5 measurements together for OA risk.

In the univariable MR, there was evidence of a detrimental effect of height, weight, BMI, waist circumference, and hip circumference on OA risk in the main IVW analyses (height: OR 1.115, 95% CI 1.054–1.180; weight: OR 1.765, 95% CI 1.650–1.889; BMI: OR 1.952, 95%CI 1.841–2.068; waist circumference: OR 2.140, 95% CI 1.994–2.296; hip circumference: OR 1.719, 95% CI 1.600–1.846), with consistent findings in sensitivity analyses **(Fig 2)**. We further validated the results of the MR analyses using the MRlap approach, which is specifically designed to correct for sample overlap in MR analyses. For most analyses, the corrected effect did not significantly differ from the observed effect, then IVW-MR estimate can be safely used. For BMI and waist circumference, which have a significant difference, corrected effects should be preferred as they should be less biased, independently of the sample overlap **(Table 2)**. The “leave-one-out analysis” plots were presented in **S16-S20 Figs in S1 File**. In an additional analysis, multivariable MR analysis was also used to assess the causal effect of single anthropometric measurement on OA while controlling for the genetic predisposition to the other anthropometric measurements. After adjusting for the confounding exposure effects by MVMR, we found the measurement mainly associated with OA was waist circumference (waist circumference: OR 1.877, 95% CI 1.286–2.739) **(Fig 2)**. Height, weight, BMI, and hip circumference did not have a statistically significant causal association with OA (height: OR 0.246, 95% CI 0.033–1.837; weight: OR 0.832, 95% CI 0.327–2.639; BMI: OR 0.670, 95%CI 0.007–2.734; hip circumference: OR 0.982, 95% CI 0.662–1.458) **(Fig 2)**.

**Table 2:** Results of MR analyses using the MRlap approach.

Exposure	Outcome	observed effect	observed 95%CI	observed P	corrected effect	corrected 95%CI	corrected P	test difference	P difference
height	OA	1.014	(1.005,1.022)	0.001	1.013	(1.004,1.022)	0.004	1.234	0.217
weight	OA	1.144	(1.129,1.159)	3.266E-91	1.144	(1.127,1.161)	9.678E-73	0.059	0.953
BMI	OA	1.188	(1.173,1.203)	2.367E-148	1.195	(1.177,1.213)	1.733E-121	-3.908	9.289E-05
waist circumference	OA	1.202	(1.183,1.220)	1.332E-121	1.213	(1.192,1.235)	4.539E-101	-4.991	5.999E-07
hip circumference	OA	1.159	(1.141,1.178)	1.336E-71	1.163	(1.142,1.184)	5.875E-58	-1.713	0.087
height	knee OA	1.008	(1.001,1.016)	0.034	1.008	(0.999,1.016)	0.073	1.881	0.060
weight	knee OA	1.139	(1.125,1.154)	2.671E-87	1.138	(1.121,1.154)	2.864E-67	1.160	0.246
BMI	knee OA	1.186	(1.171,1.202)	4.174E-149	1.192	(1.174,1.209)	4.552E-121	-2.870	0.004
waist circumference	knee OA	1.198	(1.180,1.217)	2.398E-114	1.208	(1.187,1.230)	1.082E-93	-4.360	1.301E-05
hip circumference	knee OA	1.152	(1.134,1.170)	3.246E-68	1.153	(1.133,1.174)	4.729E-54	-0.730	0.465
height	hip OA	1.008	(1.001,1.016)	0.034	1.008	(0.999,1.016)	0.070	1.023	0.306
weight	hip OA	1.139	(1.125,1.154)	2.671E-87	1.138	(1.121,1.154)	2.948E-67	1.139	0.255
BMI	hip OA	1.186	(1.171,1.202)	4.174E-149	1.192	(1.175,1.209)	2.685E-124	-2.490	0.013
waist circumference	hip OA	1.198	(1.180,1.217)	2.398E-114	1.208	(1.186,1.231)	3.994E-91	-6.545	5.955E-11
hip circumference	hip OA	1.152	(1.134,1.170)	3.246E-68	1.153	(1.133,1.174)	1.222E-54	-0.660	0.509

MR, mendelian randomization; OA, osteoarthritis; BMI, body mass index

### The causal effect of anthropometric measurements on knee OA

**Fig 3:**
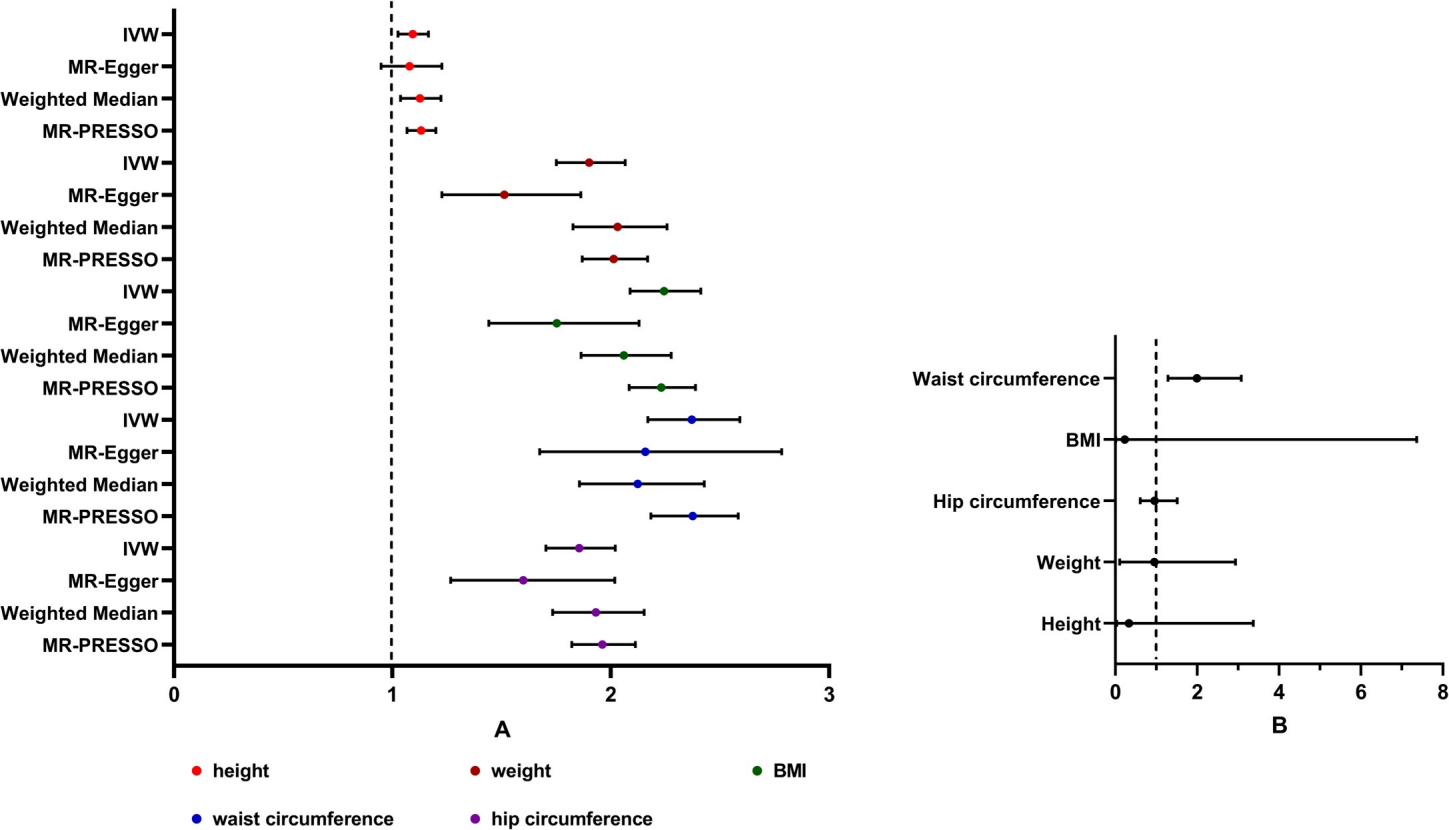
Forest plot for univariable and multivariable MR analyses of the causal effect of anthropometric measurements on knee osteoarthritis. A. Estimated causal effects of anthropometric measurements on knee osteoarthritis using univariable MR. The genetically predicted height, weight, BMI, waist circumference, and hip circumference are included. Inverse-variance weighted (IVW), MR-Egger, weighted median and MR-PRESSO represent different Mendelian randomization models. B. Estimated causal effects of anthropometric measurements on knee osteoarthritis using multivariable MR. MVMR results are from adjusting all 5 measurements together for knee OA risk.

In the univariable MR, there was evidence of a detrimental effect of height, weight, BMI, waist circumference, and hip circumference on knee OA risk in the main IVW analyses (height: OR 1.093, 95% CI 1.025–1.165; weight: OR 1.901, 95% CI 1.750–2.066; BMI: OR 2.244, 95%CI 2.088–2.412; waist circumference: OR 2.371, 95% CI 2.170–2.591; hip circumference: OR 1.855, 95% CI 1.703–2.020), with consistent findings in sensitivity analyses **(Fig 3)**. The validated results of the MR analyses using the MRlap approach were presented in **Table 2**. The “leave-one-out analysis” plots were presented in **S21-S25 Figs in S1 File**. However, after adjusting for the confounding exposure effects by MVMR, we found the measurement mainly associated with knee OA was waist circumference (waist circumference: OR 1.989, 95% CI 1.286–3.074) **(Fig 3)**. Height, weight, BMI, and hip circumference did not have a statistically significant causal association with OA (height: OR 0.332, 95% CI 0.033–3.365; weight: OR 0.952, 95% CI 0.106–2.935; BMI: OR 0.228, 95%CI 0.007–7.359; hip circumference: OR 0.958, 95% CI 0.608–1.510) **(Fig 3)**.

### The causal effect of anthropometric measurements on hip OA

**Fig 4:**
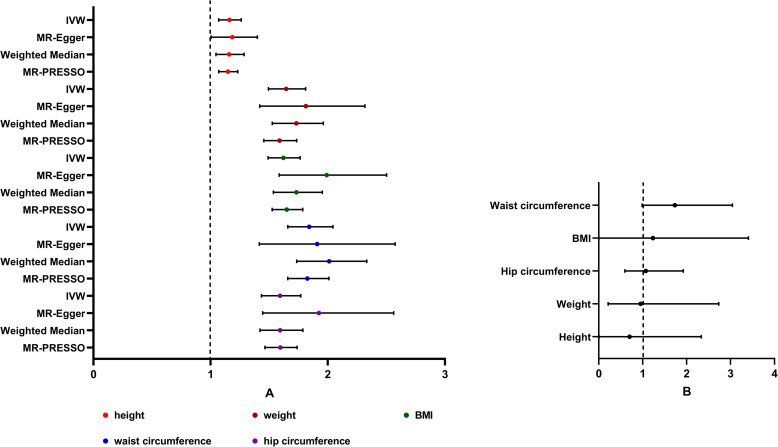
Forest plot for univariable and multivariable MR analyses of the causal effect of anthropometric measurements on hip osteoarthritis. A. Estimated causal effects of anthropometric measurements on hip osteoarthritis using univariable MR. The genetically predicted height, weight, BMI, waist circumference, and hip circumference are included. Inverse-variance weighted (IVW), MR-Egger, weighted median and MR-PRESSO represent different Mendelian randomization models. B. Estimated causal effects of anthropometric measurements on hip osteoarthritis using multivariable MR. MVMR results are from adjusting all 5 measurements together for hip OA risk.

In the univariable MR, there was evidence of a detrimental effect of height, weight, BMI, waist circumference, and hip circumference on hip OA risk in the main IVW analyses (height: OR 1.161, 95% CI 1.069–1.261; weight: OR 1.645, 95% CI 1.493–1.811; BMI: OR 1.621, 95%CI 1.490–1.764; waist circumference: OR 1.841, 95% CI 1.658–2.044; hip circumference: OR 1.593, 95% CI 1.434–1.769), with consistent findings in sensitivity analyses **(Fig 4)**. The validated results of the MR analyses using the MRlap approach were presented in **Table 2**. The “leave-one-out analysis” plots were presented in **S26-S30 Figs in S1 File**. However, after adjusting for the confounding exposure effects by MVMR, we found that the associations between anthropometric measurements and hip OA were not statistically significant (height: OR 0.702, 95% CI 0.006–2.334; weight: OR 0.952, 95% CI 0.212–2.732; BMI: OR 1.232, 95%CI 0.004–3.406; waist circumference: OR 1.732, 95% CI 0.986–3.041; hip circumference: OR 1.069, 95% CI 0.594–1.924) **(Fig 4)**.

## Discussion

Our work uses large-scale GWAS data to investigate the effect of anthropometric measurements on OA risk within the MR framework. After analysis, we found that waist circumference was associated with total OA; therefore, we further explored the relationship between the two components that make up the total OA (knee and hip) and waist circumference separately. We found that only knee OA was associated with waist circumference. We therefore concluded that the causal association between anthropometric measurements and OA was mainly focused on waist circumference and knee OA.

Consistent with our results, increased waist circumference has been associated with progression of OA in both longitudinal epidemiological and cross-sectional research [43, 44]. Compared to other measurements, why is waist circumference associated with OA more significant? The reason may be that waist circumference is more sufficient to distinguish obese people with an elevated risk of health problems in comparison with other anthropometric measurements. Obesity comes in a variety of forms. Differences in body shape due to different fat distribution have been observed for a long time. And this observation was also confirmed in imaging. MRI and CT have uncovered differences between subjects in the proportion of adipose tissue lodged in the abdominal cavity: certain obese subjects have little visceral adipose tissue, while others with the same total fat mass, have a greater amount of visceral adipose tissue. In 1947, Jean Vague introduced the waist circumference measurement to differentiate between central obesity (visceral, ectopic adipose tissue) and peripheral obesity (subcutaneous adipose tissue). In subsequent studies, it was found that central obesity, which is closely related to waist circumference, was damaging to human health. This visceral adipose tissue can induce a range of inflammatory responses and further lead to a variety of diseases. A meta-analysis on more than 650,000 individuals concluded that no matter the BMI (normal, overweight, obese; BMI ranging from 20 to 50 kg/m2), an increase in waist circumference results in a significant and identical increase in the mortality risk [45]. Similarly, several studies suggest that an elevated waist circumference, regardless of BMI, is also a risk factor for OA [16]. Secondly, from a biomechanical perspective, excess waist circumference impairs postural stability in adults due to an anterior shift in the center of mass [46]. Previous studies show that waist circumference is also associated with atypical walking patterns (i.e., decreased walking endurance and slow walking speed) in adults [47]. Higher waist circumference predicts a decline in walking speed in adults aged 55 to 74 years old [48]. Continual loading on the joints and the anterior shift in the center of mass can accelerate the rate of OA (especially for knee OA) progression and have detrimental effects on patients’ functional performance. Therefore, the distribution of body mass (as captured by waist circumference) may be associated with OA above and beyond the effects imposed by the overall mass [49]. The results suggest that future research on gait and obesity from a biomechanical perspective would benefit from considering body mass distribution as a main factor. Including waist circumference as a possible variable would augment our knowledge of how body mass distribution influences function. Different from our current findings, previous studies have identified a positive association between various anthropometric measurements and risk of OA [18, 20, 50]. This discrepancy may be attributable to confounders in observational studies as well as the correlation and pleiotropy of exposures in univariable MR.

There are limitations to our study. Firstly, due to limited resources, we are unable to obtain the most recent individual-level statistics. Secondly, anthropometric measures may differentially contribute to weight-bearing vs. non-weight bearing joints. Unfortunately, we are unable to obtain publicly available GWAS for hand OA at present. Thirdly, the summary GWAS data only includes people of European lineage, so our findings may not be completely representative of the entire population. More studies should be conducted to verify the applicability of these results to other ethnicities.

In summary, the study provides evidence supporting a detrimental effect of waist circumference on knee OA risk. This suggests we should pay more attention to the detrimental effects of factors increasing waist circumference (like central obesity) on OA risk. Weight reduction targeting waist circumference should be highlighted in older adults at risk for OA.

## Supporting information

S1 File S1-S30 Figs are included in the file. (ZIP)

S2 File S1-S18 Tables are included in the file. (ZIP)
