# Supplementary material for: Causal associations of anthropometric measurements with osteoarthritis: A Mendelian randomization study
Source: PLoS One. 2023 Jan 30;18(1):e0279198. doi: 10.1371/journal.pone.0279198 (PMC9886244; doi:10.1371/journal.pone.0279198)
Supplement: S1 File — (ZIP) [file pone.0279198.s001.zip › Supporting Figs.docx]

***Supporting Figs***

Causal Associations of Anthropometric Measurements with Osteoarthritis: A Mendelian Randomization Study


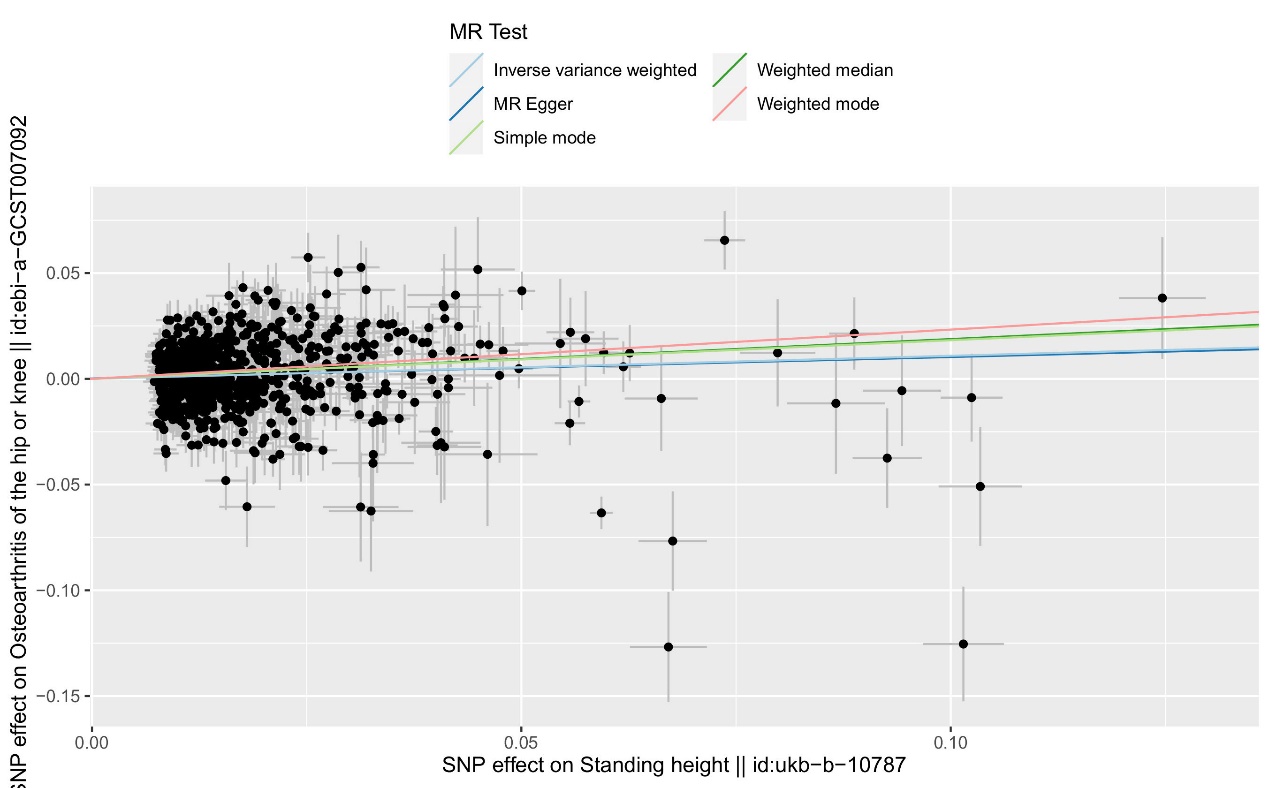
**Supplementary Figure 1**. A scatterplot of variant-height (x-axis) and variant-OA risk (y-axis) association estimates for height instruments in univariable MR analysis. The slope of each line corresponds to the effect size obtained by the corresponding MR method.


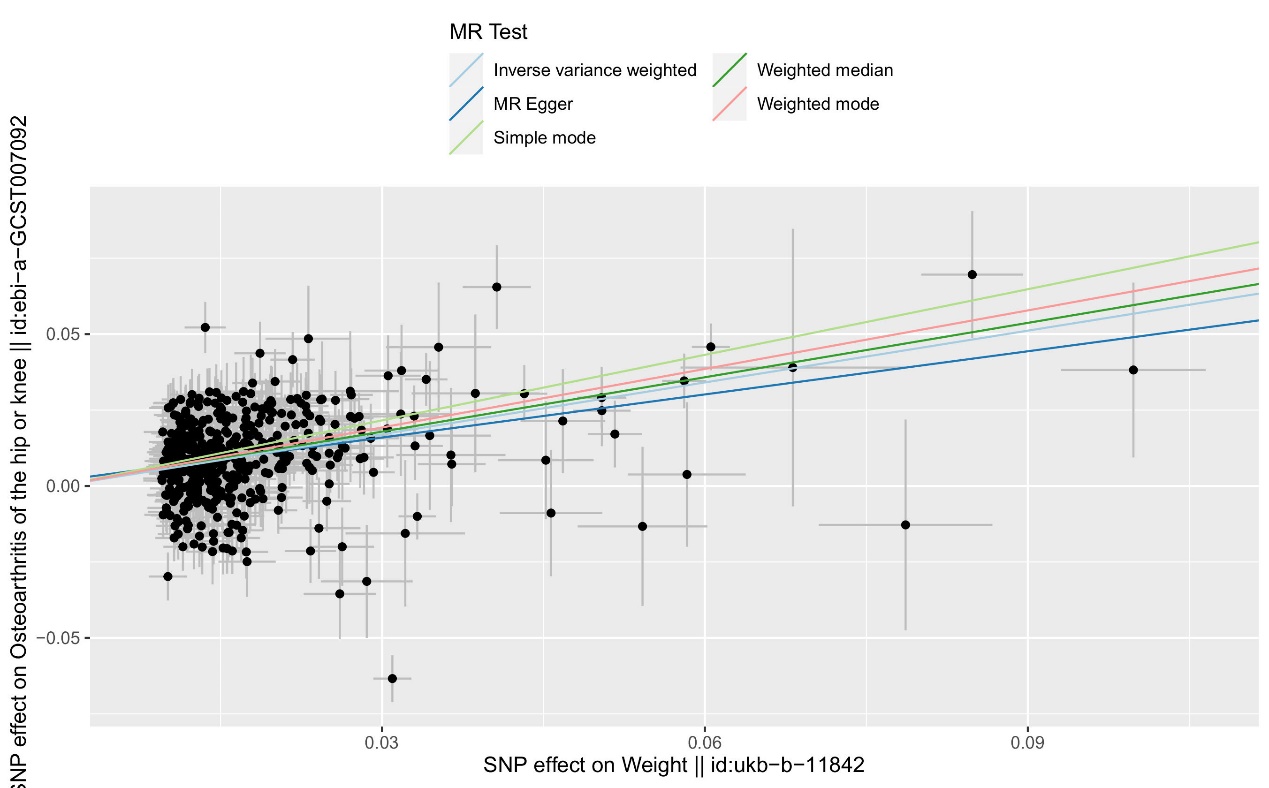


**Supplementary Figure 2.** A scatterplot of variant-weight (x-axis) and variant-OA risk (y-axis) association estimates for weight instruments in univariable MR analysis. The slope of each line corresponds to the effect size obtained by the corresponding MR method.


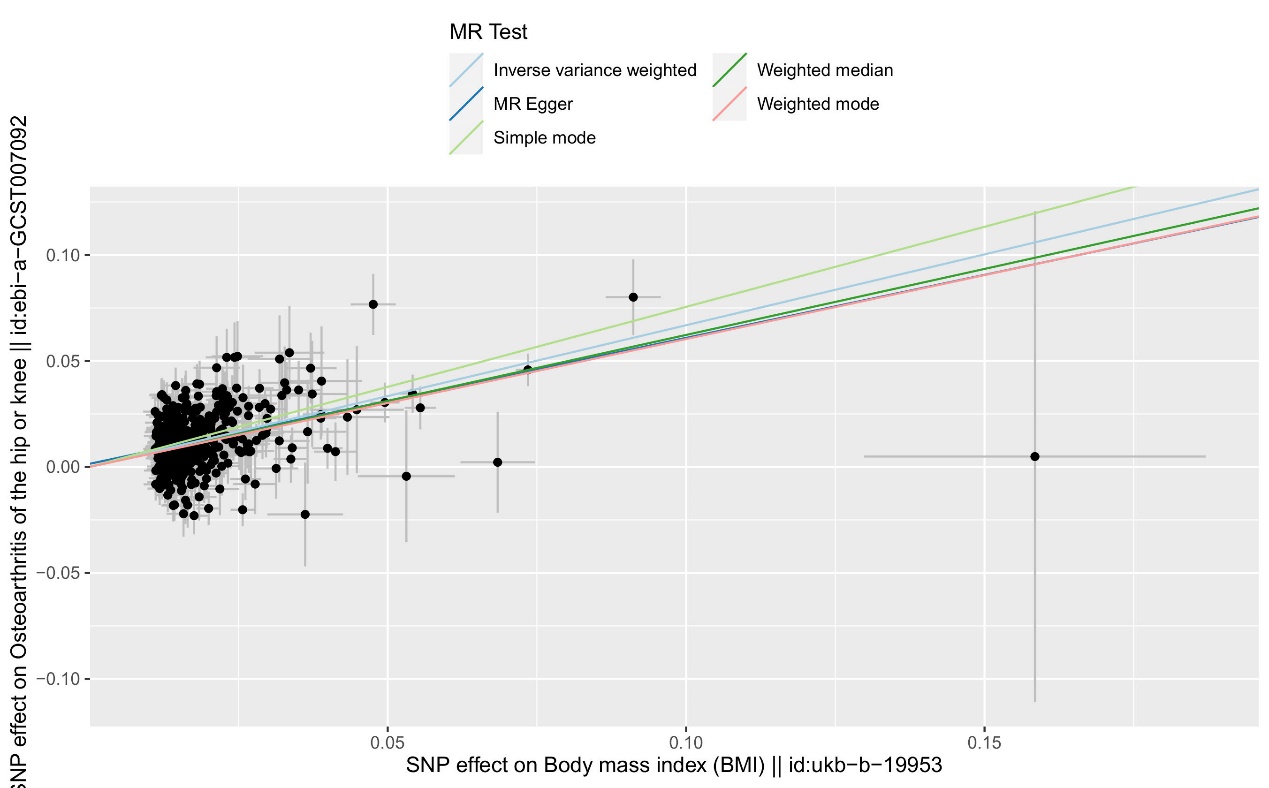


**Supplementary Figure 3**. A scatterplot of variant-BMI (x-axis) and variant-OA risk (y-axis) association estimates for BMI instruments in univariable MR analysis. The slope of each line corresponds to the effect size obtained by the corresponding MR method


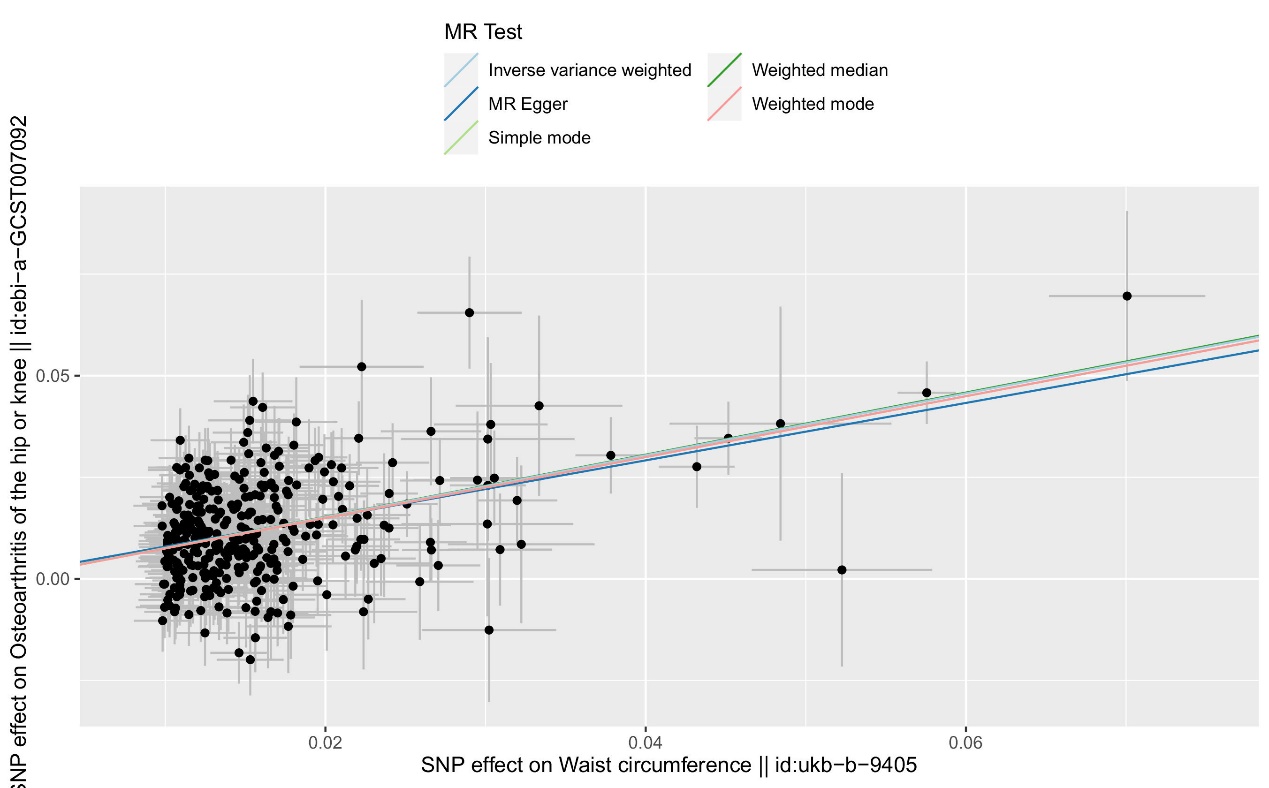


**Supplementary Figure 4**. A scatterplot of variant-waist circumference (x-axis) and variant-OA risk (y-axis) association estimates for waist circumference instruments in univariable MR analysis. The slope of each line corresponds to the effect size obtained by the corresponding MR method.


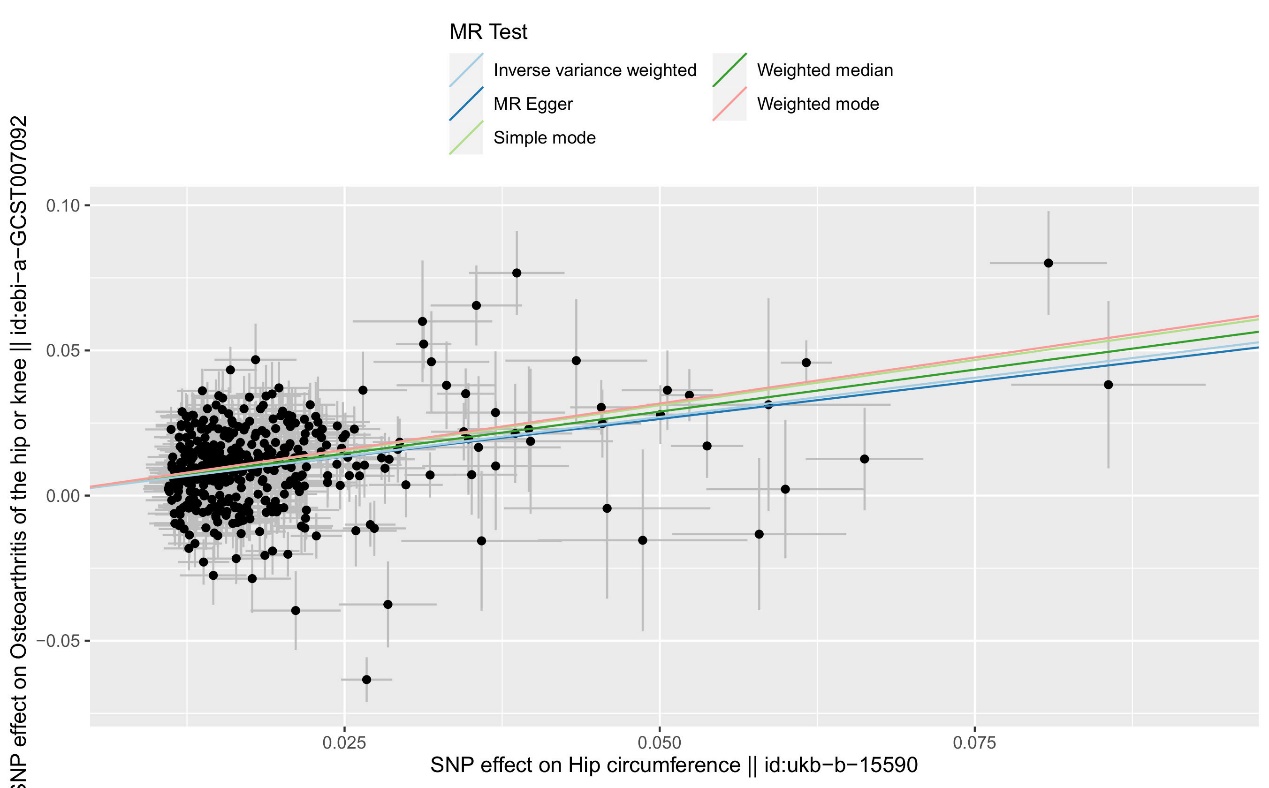


**Supplementary Figure 5**. A scatterplot of variant-hip circumference (x-axis) and variant-OA risk (y-axis) association estimates for hip circumference instruments in univariable MR analysis. The slope of each line corresponds to the effect size obtained by the corresponding MR method.


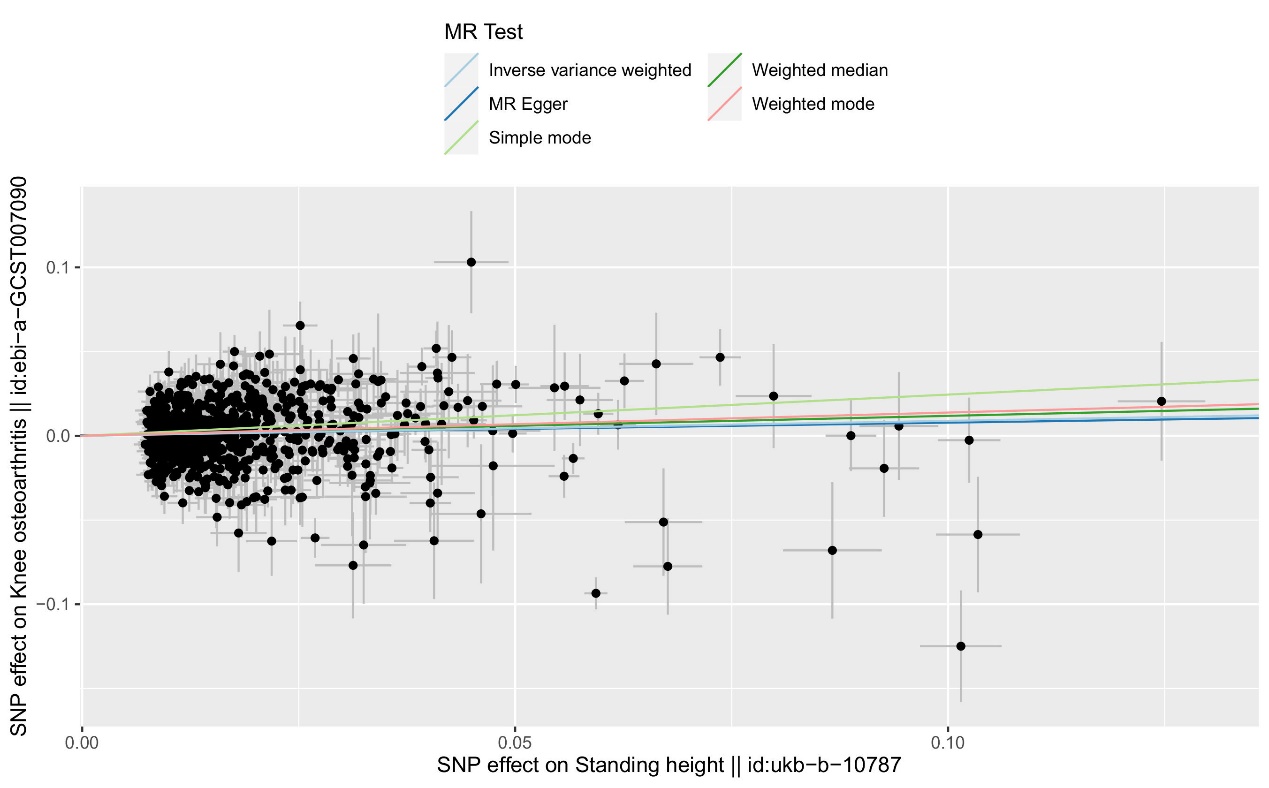


**Supplementary Figure 6**. A scatterplot of variant-height (x-axis) and variant-knee OA risk (y-axis) association estimates for height instruments in univariable MR analysis. The slope of each line corresponds to the effect size obtained by the corresponding MR method


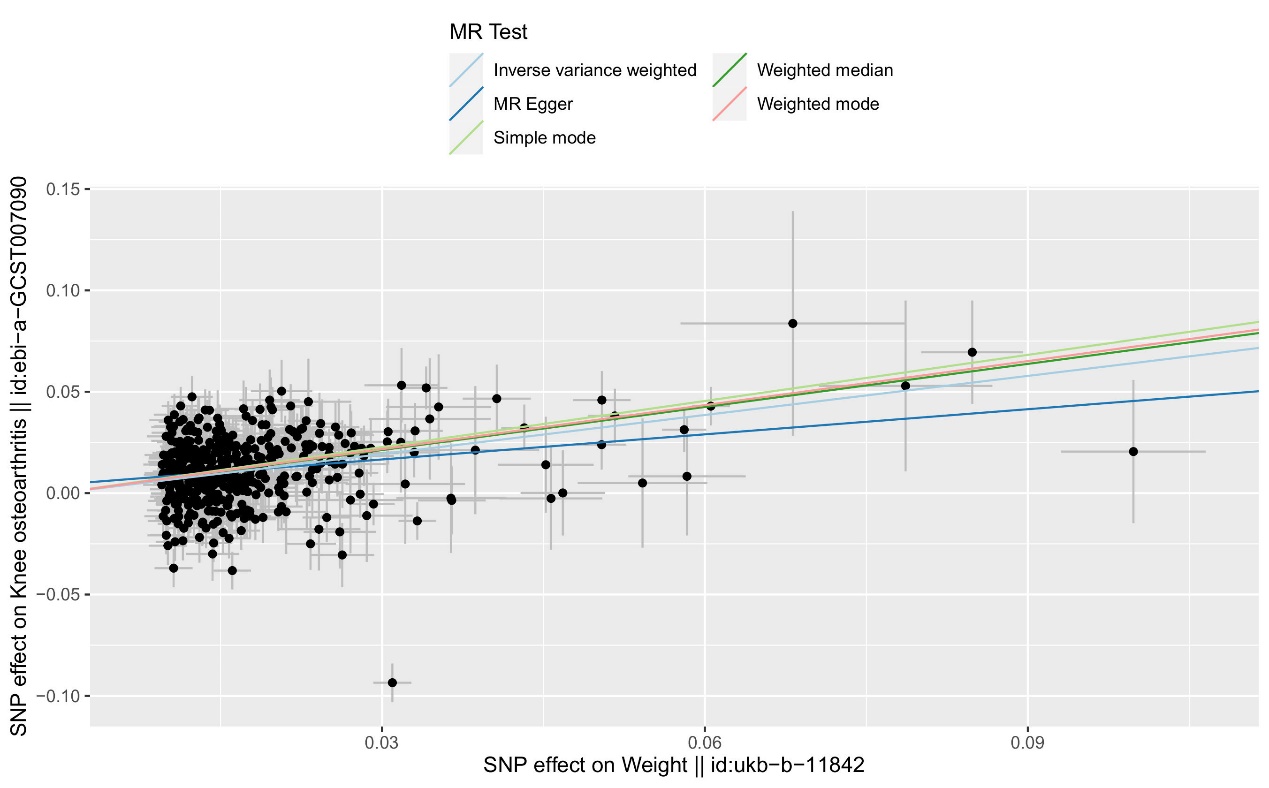


**Supplementary Figure 7**. A scatterplot of variant-weight (x-axis) and variant-knee OA risk (y-axis) association estimates for weight instruments in univariable MR analysis. The slope of each line corresponds to the effect size obtained by the corresponding MR method.


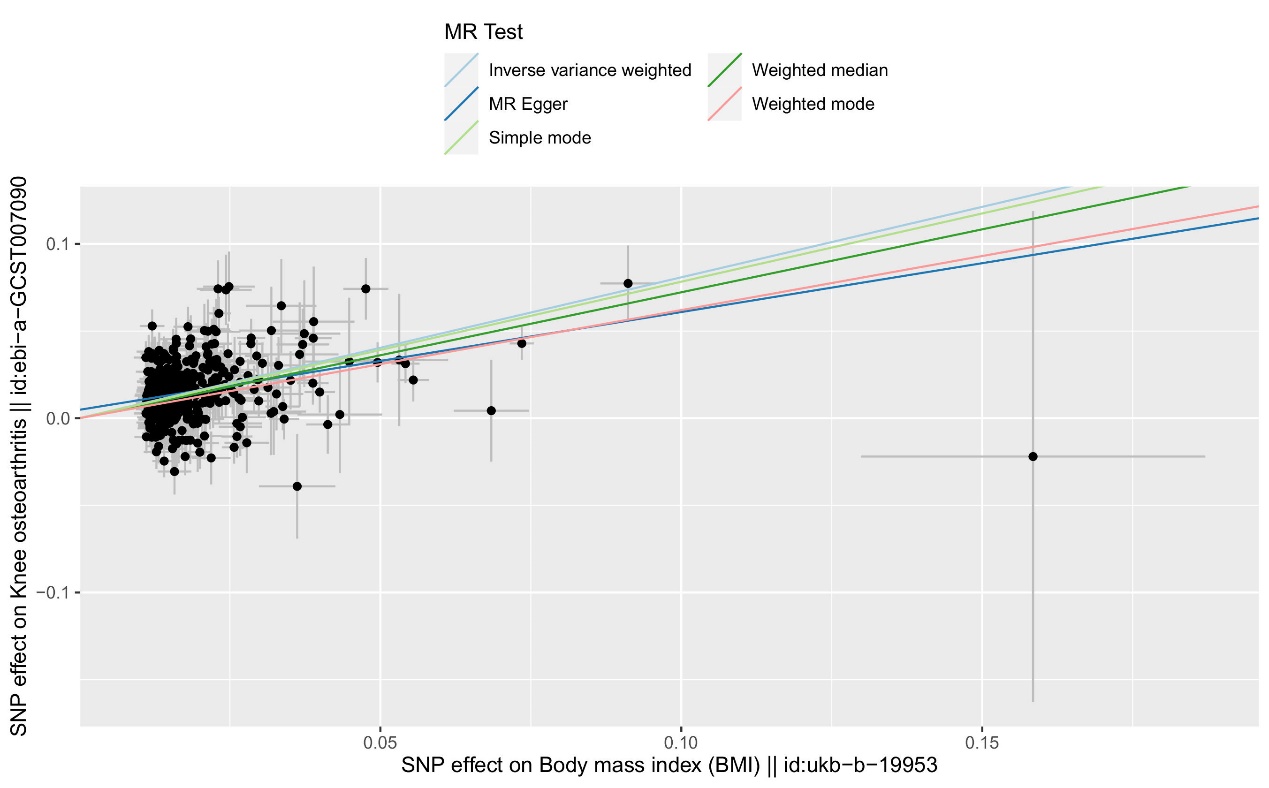


**Supplementary Figure 8**. A scatterplot of variant- BMI (x-axis) and variant-knee OA risk (y-axis) association estimates for BMI instruments in univariable MR analysis. The slope of each line corresponds to the effect size obtained by the corresponding MR method.


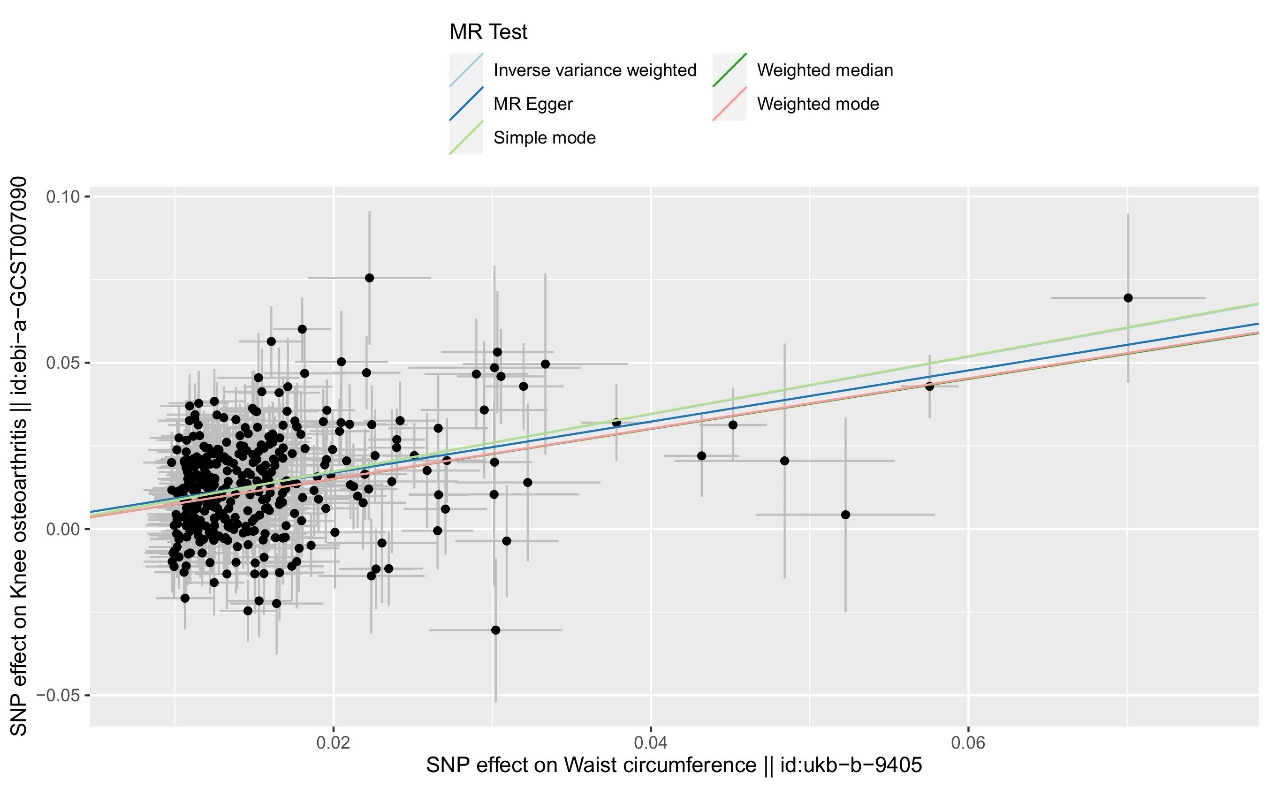
 **Supplementary Figure 9**. A scatterplot of waist circumference (x-axis) and variant-knee OA risk (y-axis) association estimates for waist circumference instruments in univariable MR analysis. The slope of each line corresponds to the effect size obtained by the corresponding MR method.


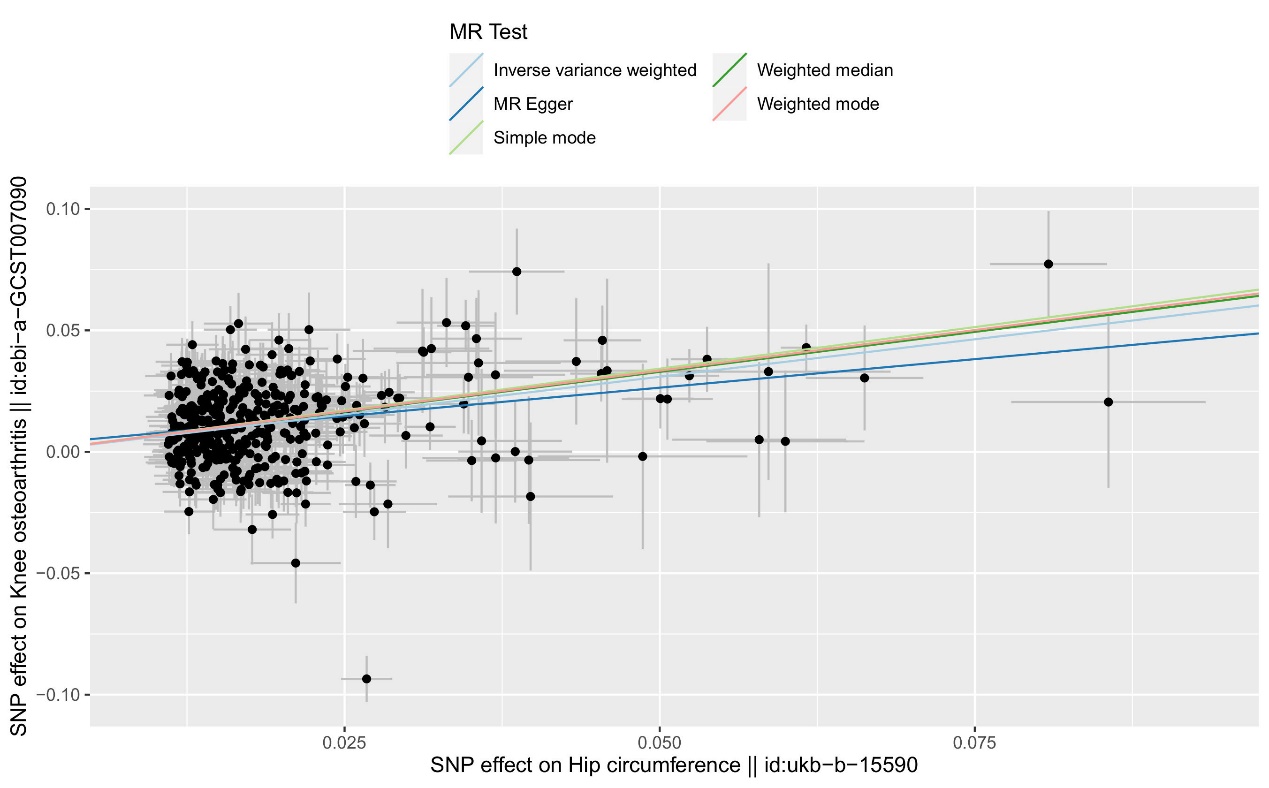
 **Supplementary Figure 10**. A scatterplot of variant- hip circumference (x-axis) and variant-knee OA risk (y-axis) association estimates for hip circumference instruments in univariable MR analysis. The slope of each line corresponds to the effect size obtained by the corresponding MR method.


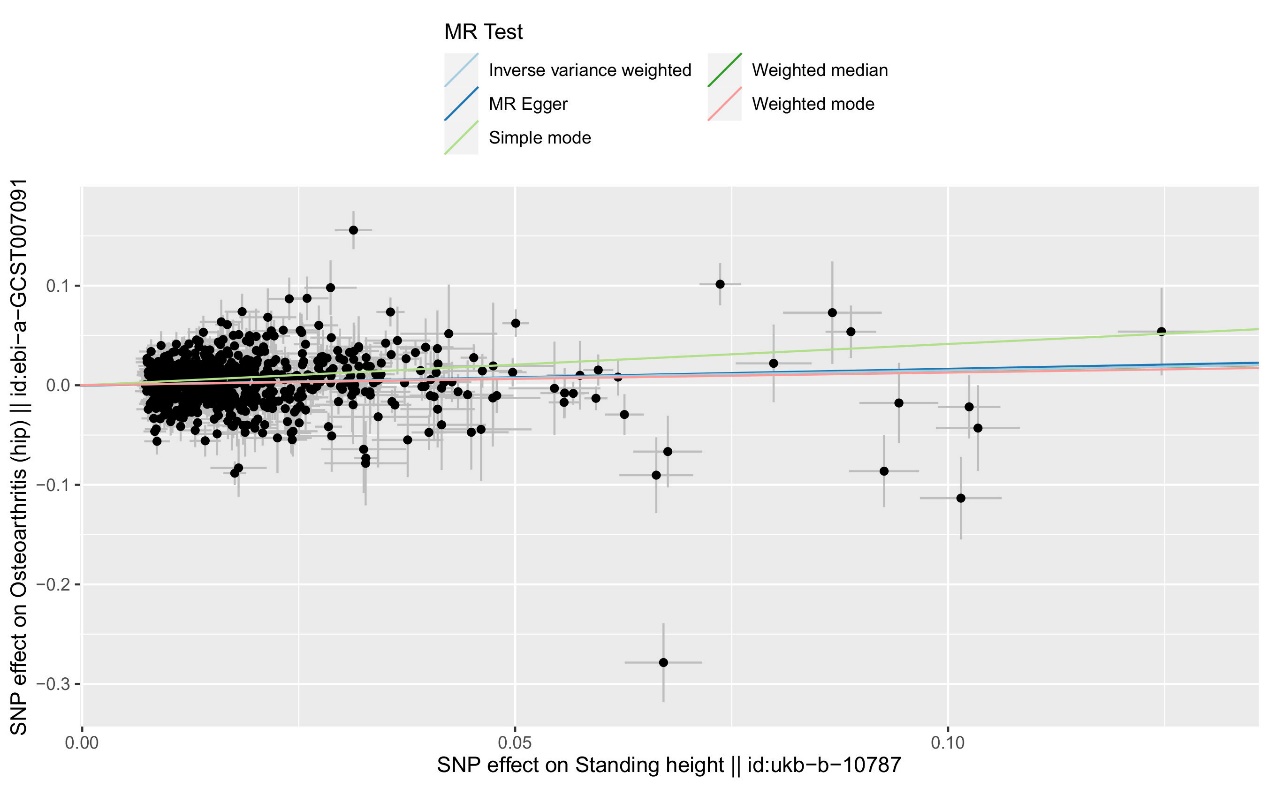
 **Supplementary Figure 11**. A scatterplot of variant-height (x-axis) and variant-hip OA risk (y-axis) association estimates for height instruments in univariable MR analysis. The slope of each line corresponds to the effect size obtained by the corresponding MR method.


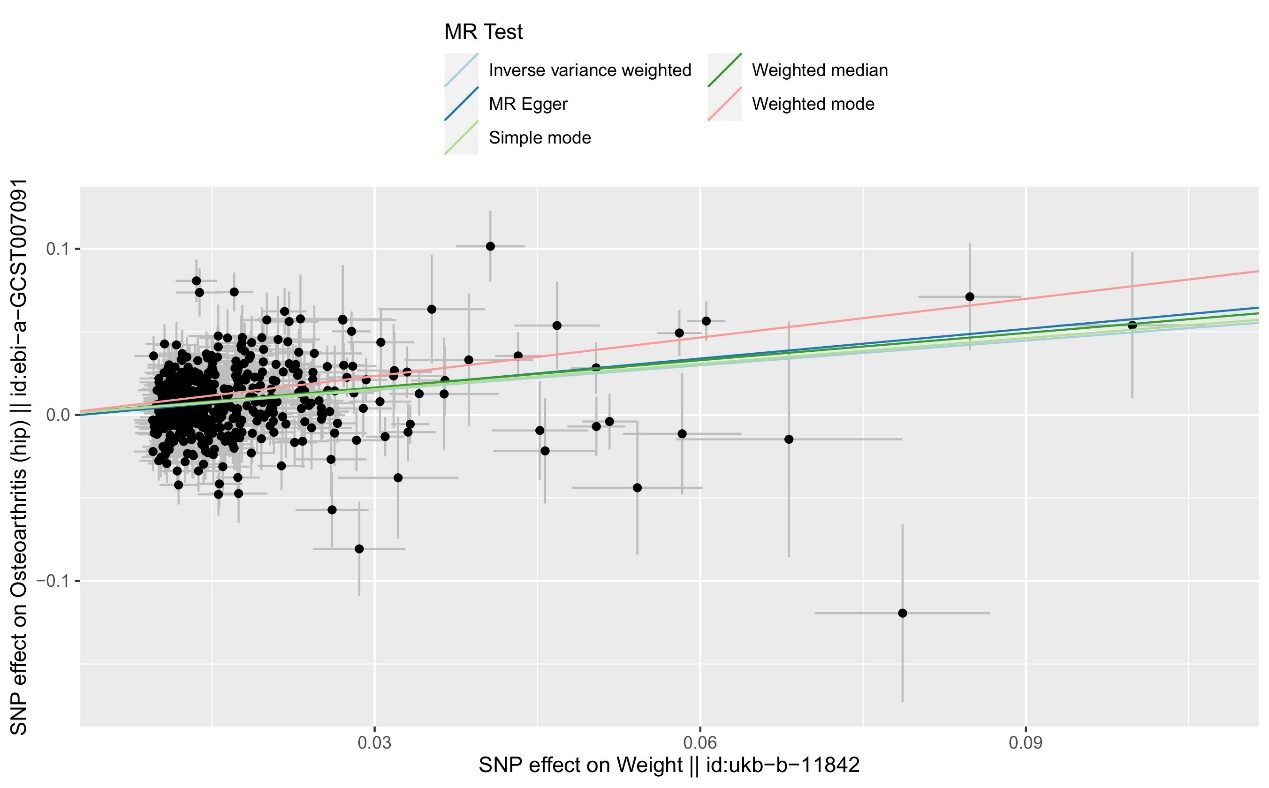
 **Supplementary Figure 12**. A scatterplot of variant-weight (x-axis) and variant-hip OA risk (y-axis) association estimates for weight instruments in univariable MR analysis. The slope of each line corresponds to the effect size obtained by the corresponding MR method.


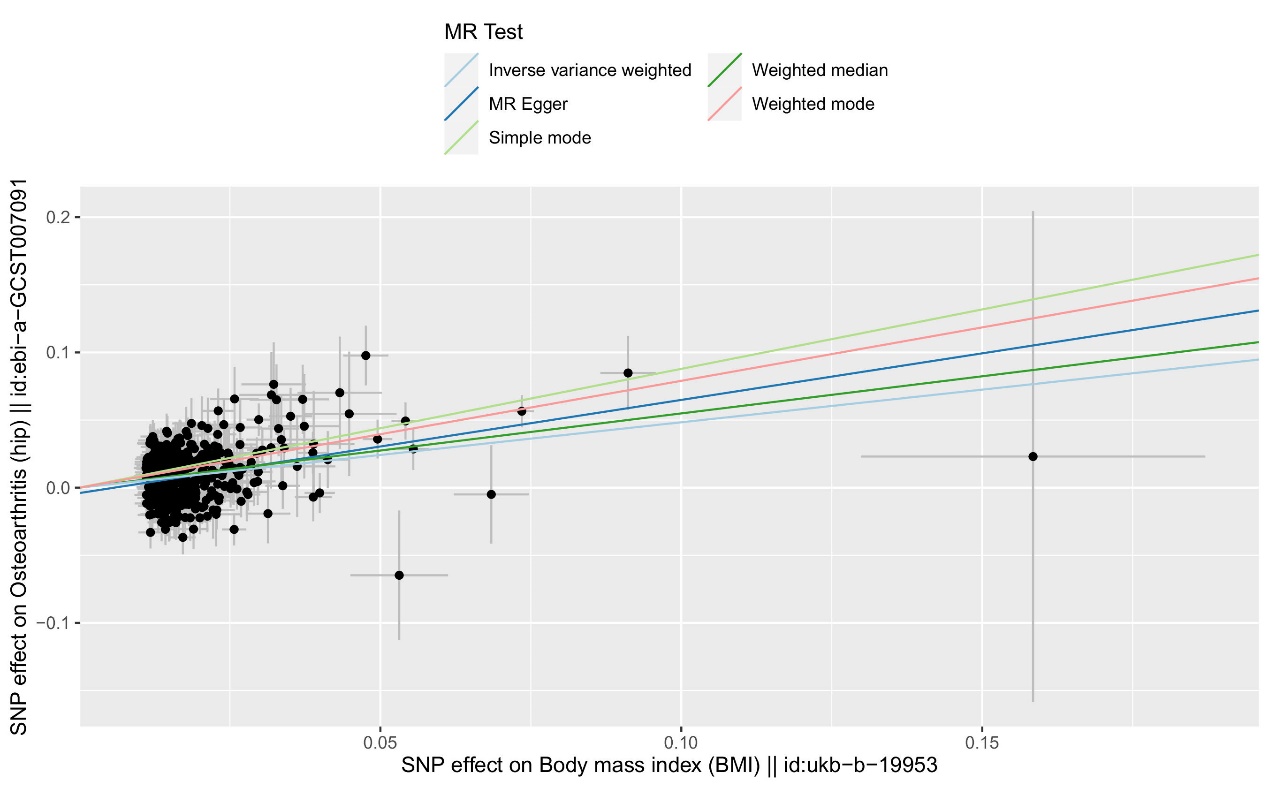
 **Supplementary Figure 13**. A scatterplot of variant- BMI (x-axis) and variant-hip OA risk (y-axis) association estimates for BMI instruments in univariable MR analysis. The slope of each line corresponds to the effect size obtained by the corresponding MR method.


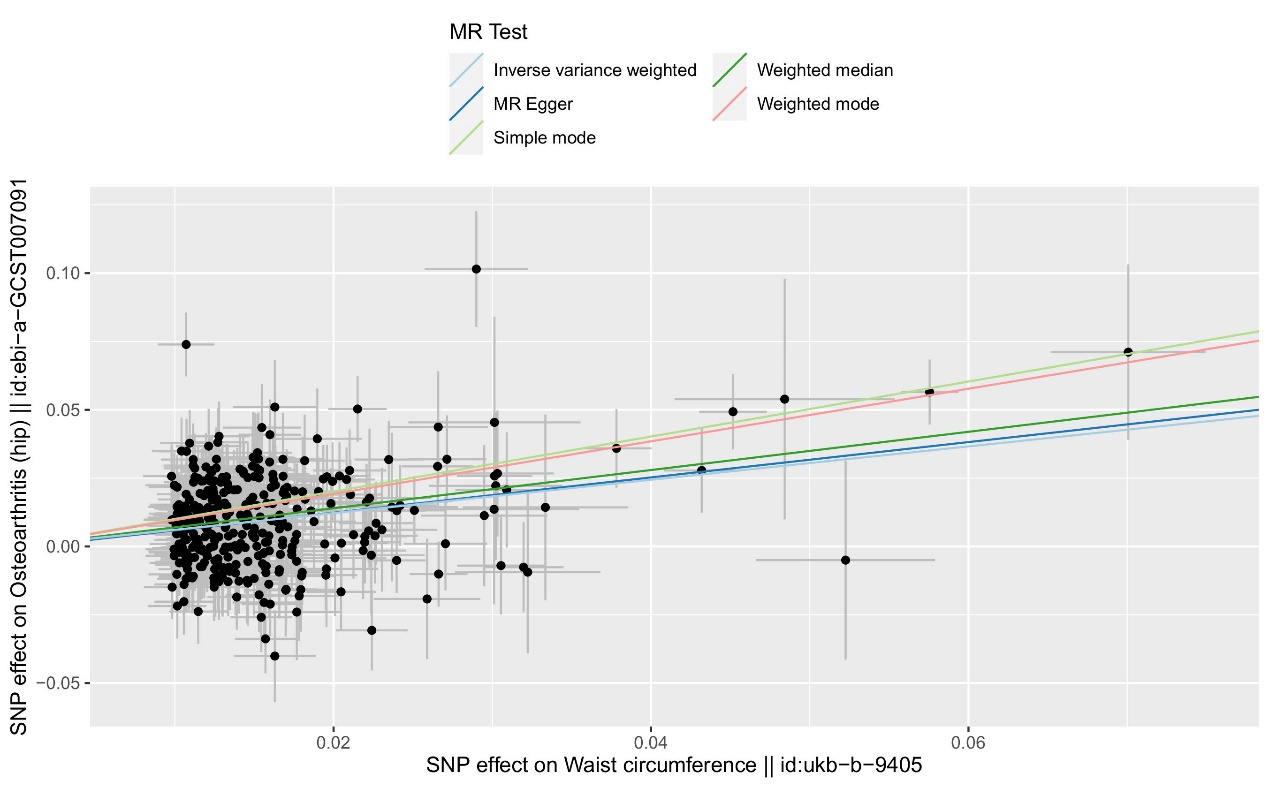
 **Supplementary Figure 14**. A scatterplot of variant- waist circumference (x-axis) and variant-hip OA risk (y-axis) association estimates for waist circumference instruments in univariable MR analysis. The slope of each line corresponds to the effect size obtained by the corresponding MR method.


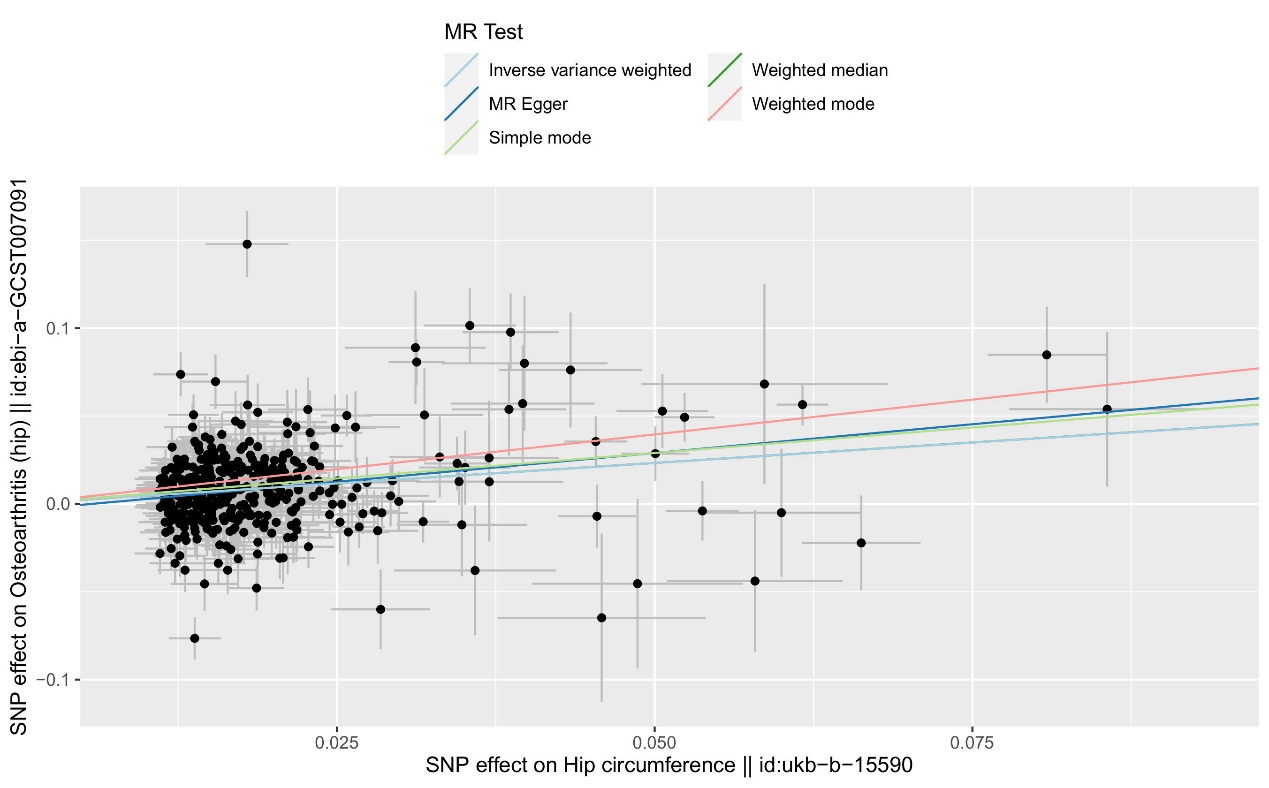
 **Supplementary Figure 15**. A scatterplot of variant- hip circumference (x-axis) and variant-hip OA risk (y-axis) association estimates for hip circumference instruments in univariable MR analysis. The slope of each line corresponds to the effect size obtained by the corresponding MR method.


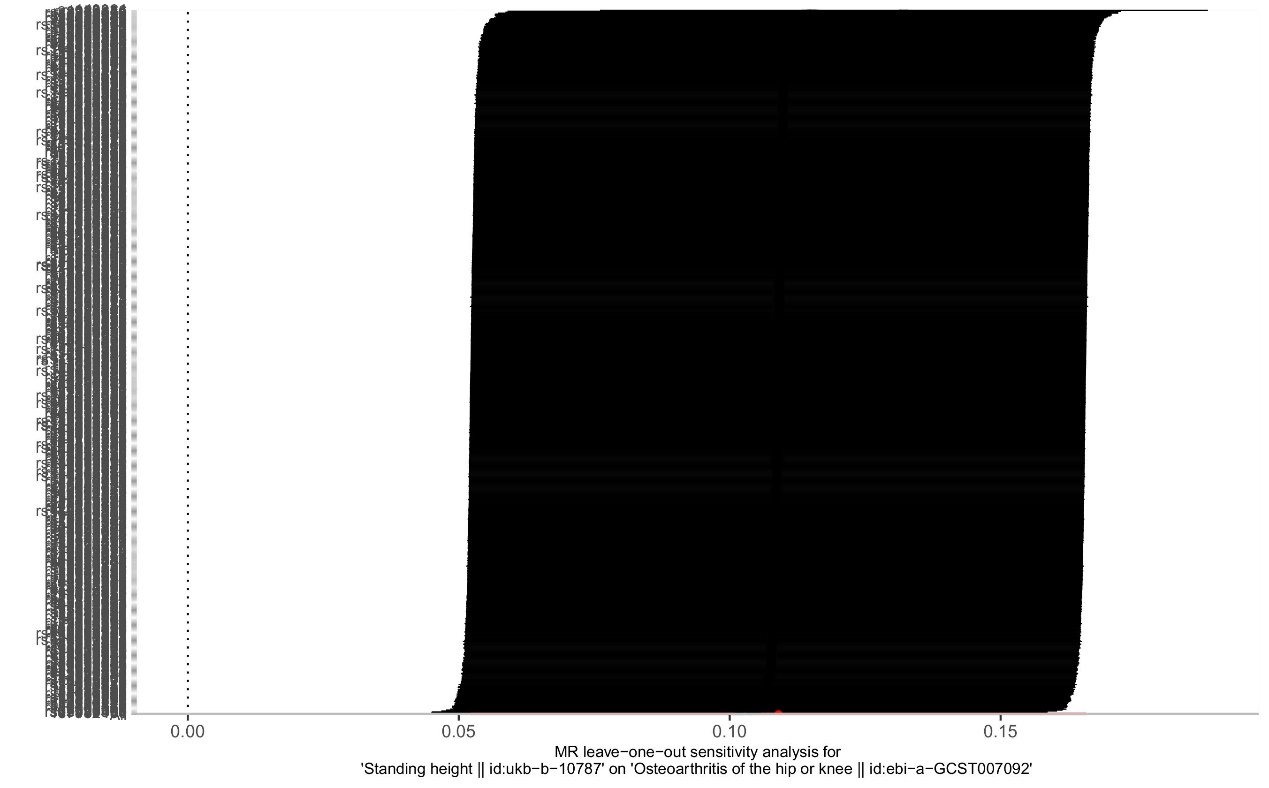


**Supplementary Figure 16**. A “leave-one-out analysis” plot of height instruments for OA in univariable MR analysis.


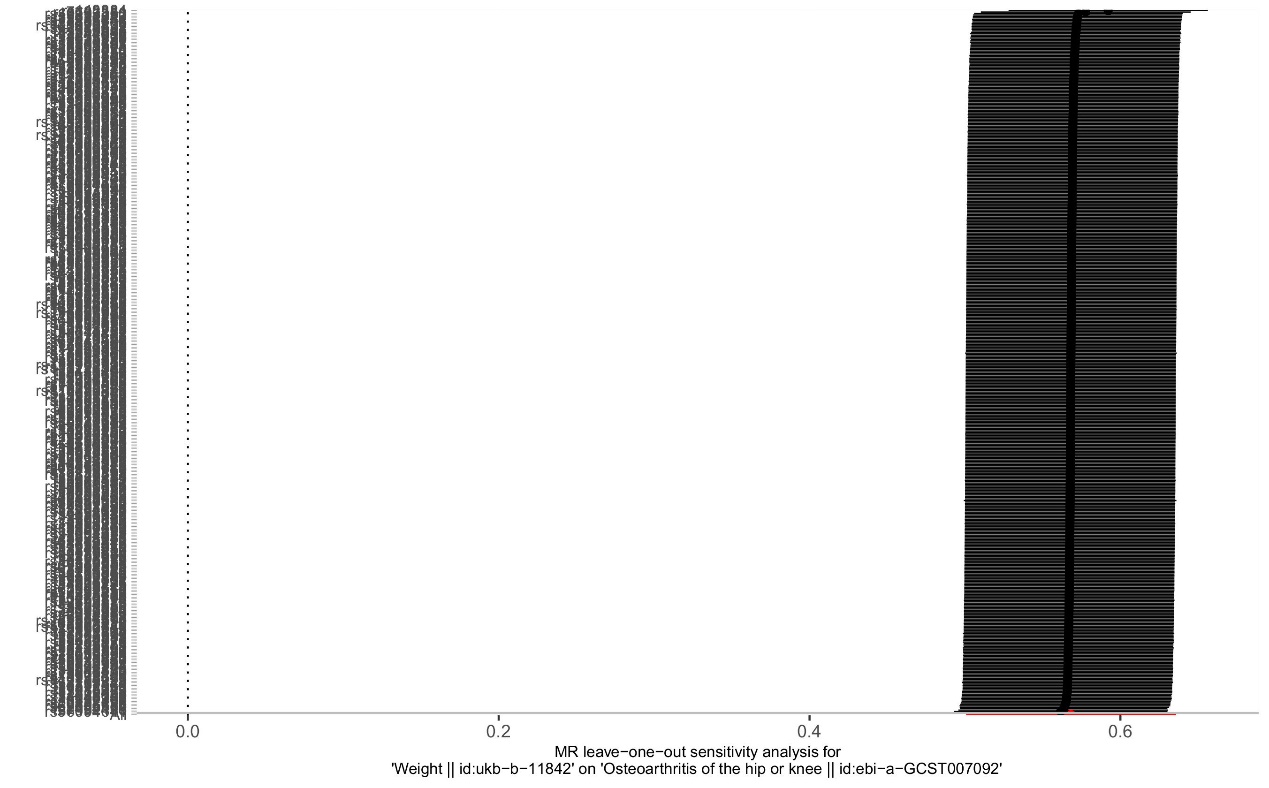


**Supplementary Figure 17**. A “leave-one-out analysis” plot of weight instruments for OA in univariable MR analysis.


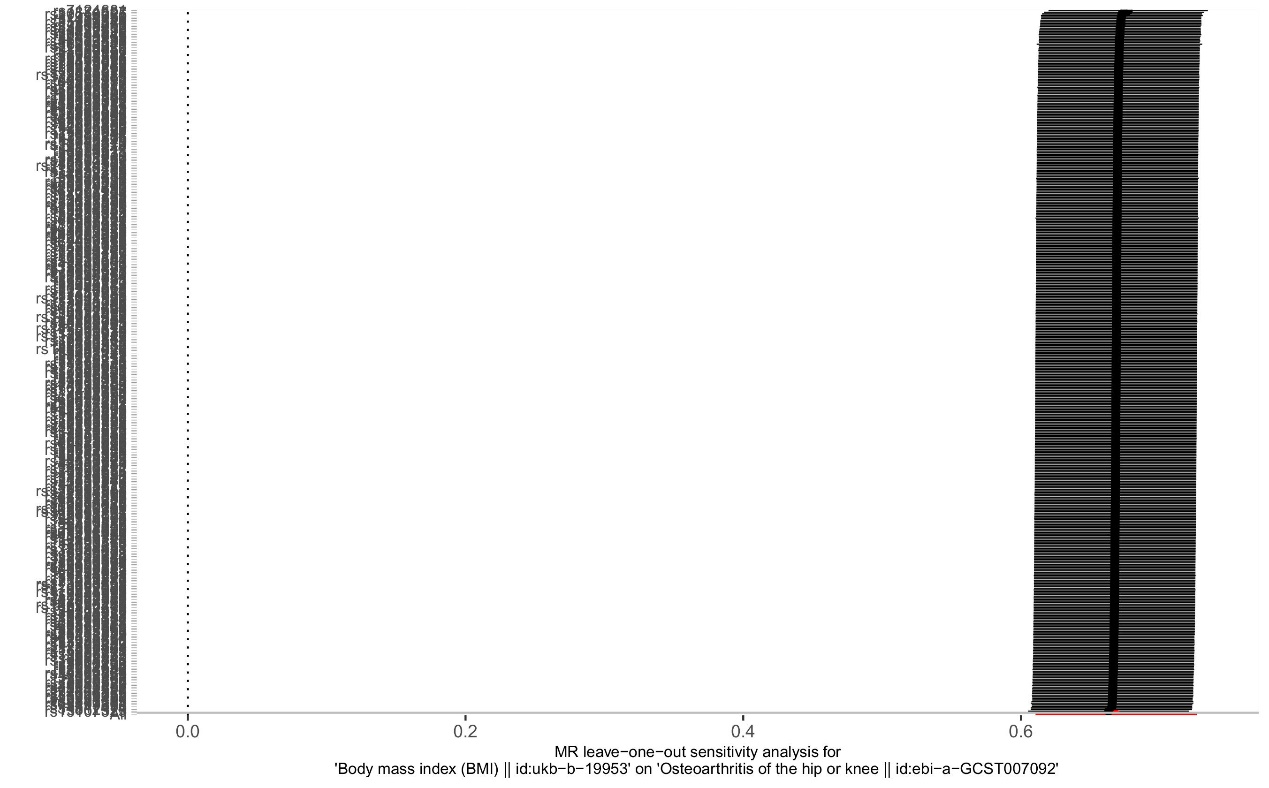


**Supplementary Figure 18**. A “leave-one-out analysis” plot of BMI instruments for OA in univariable MR analysis.


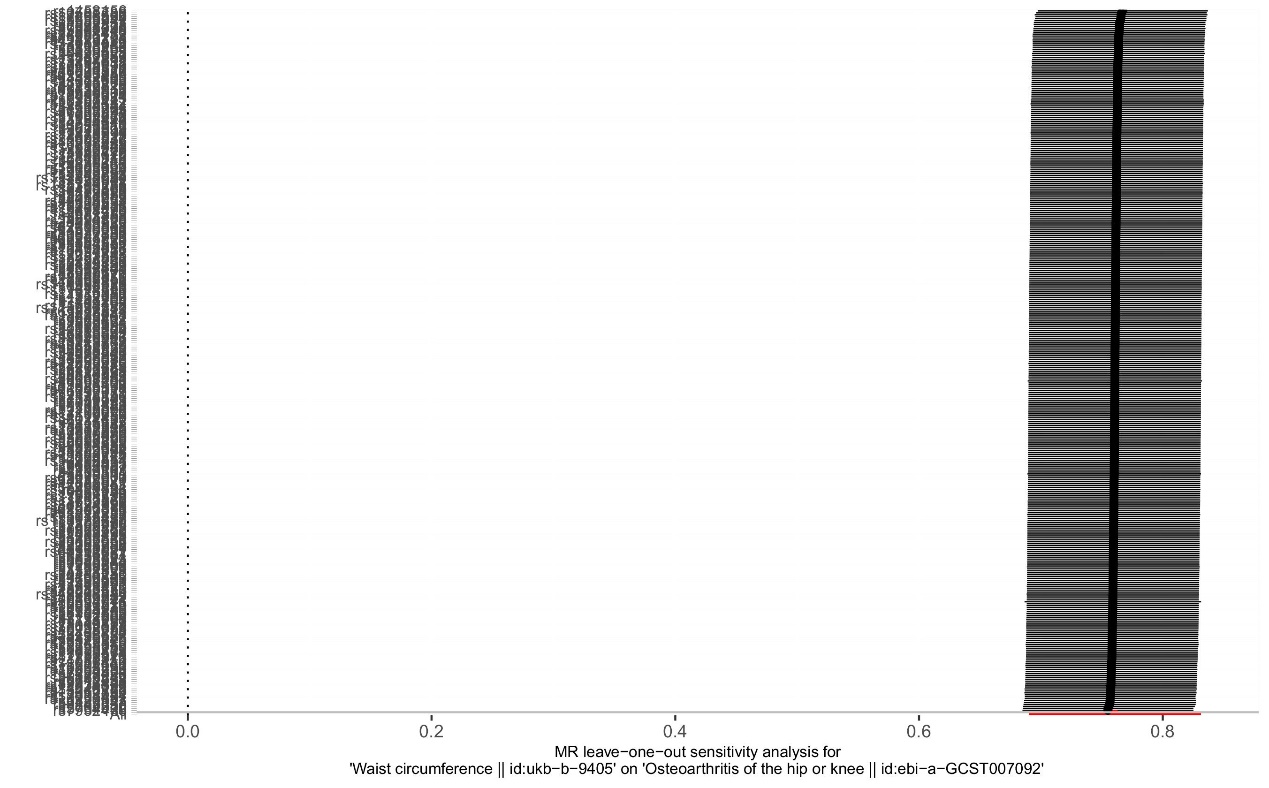


**Supplementary Figure 19**. A “leave-one-out analysis” plot of waist circumference instruments for OA in univariable MR analysis.


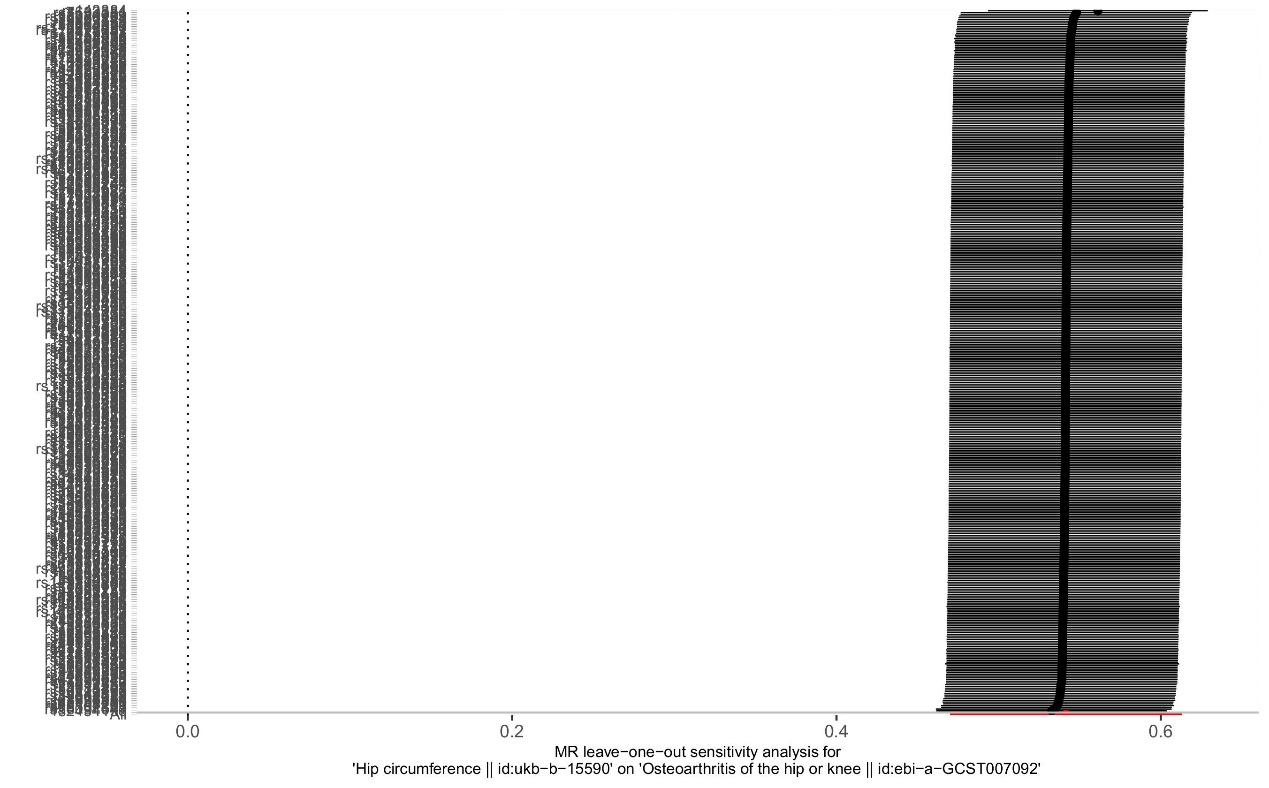


**Supplementary Figure 20**. A “leave-one-out analysis” plot of hip circumference instruments for OA in univariable MR analysis.


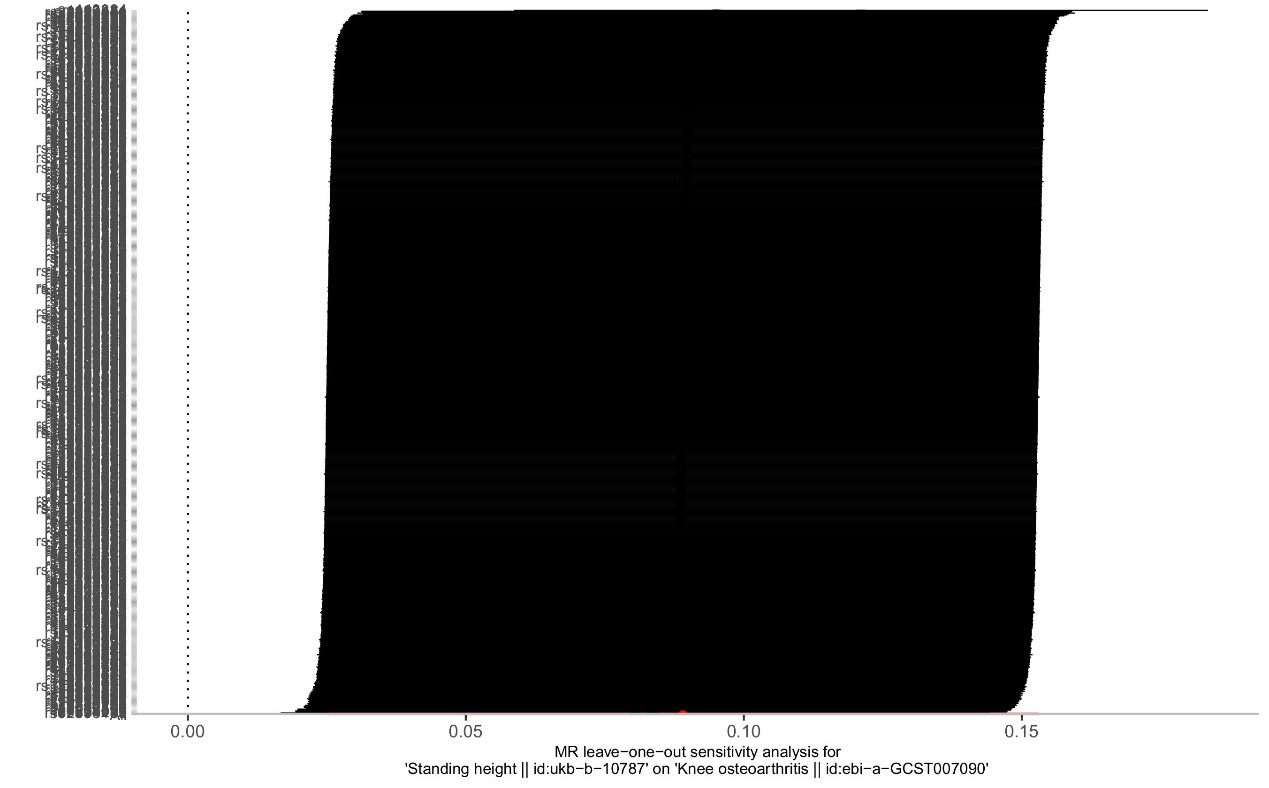


**Supplementary Figure 21**. A “leave-one-out analysis” plot of height instruments for knee OA in univariable MR analysis.


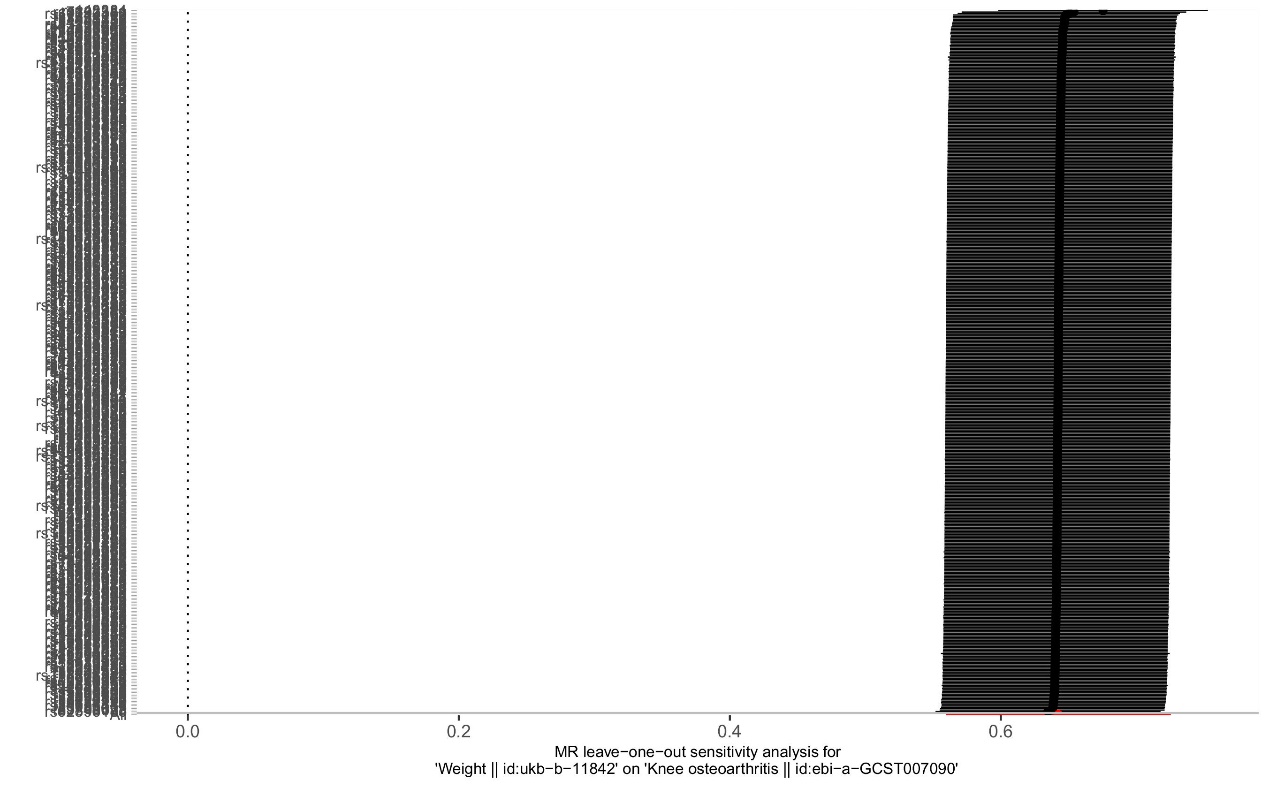
 **Supplementary Figure 22**. A “leave-one-out analysis” plot of weight instruments for knee OA in univariable MR analysis.


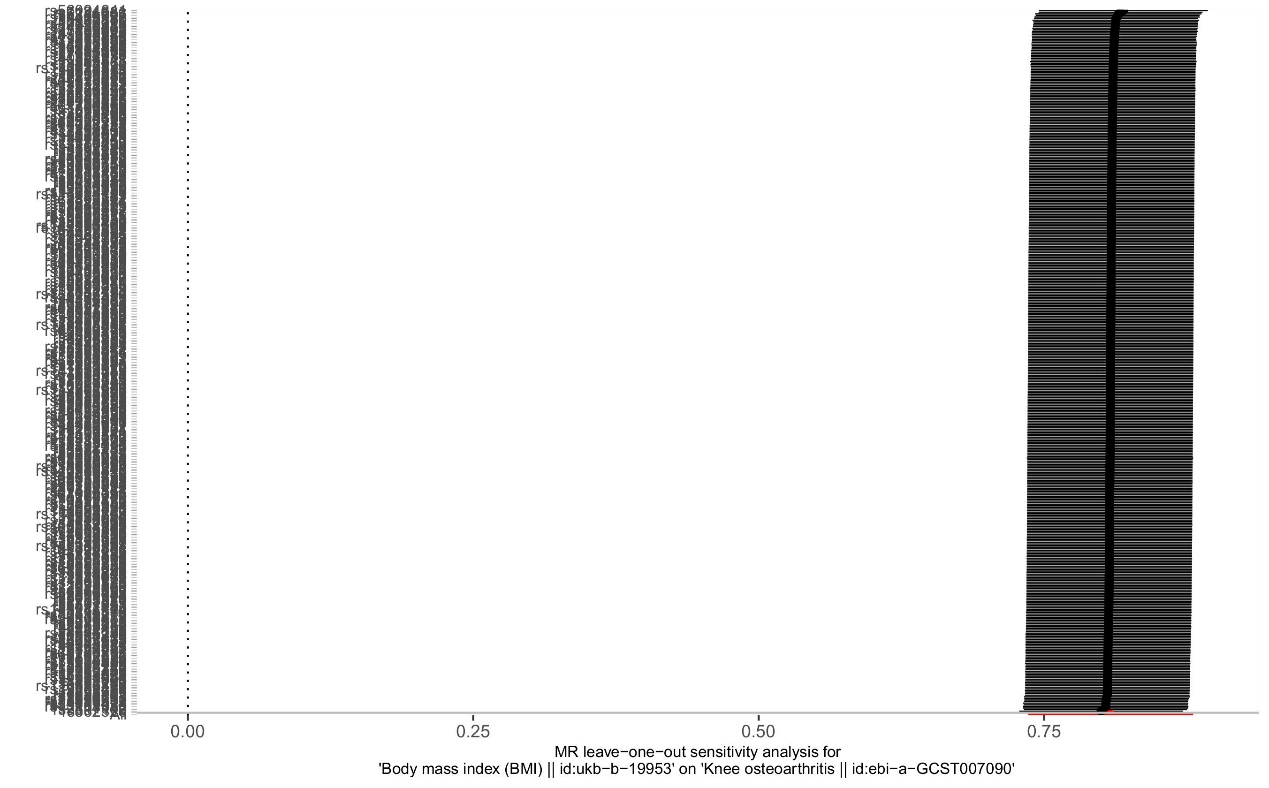
 **Supplementary Figure 23**. A “leave-one-out analysis” plot of BMI instruments for knee OA in univariable MR analysis.


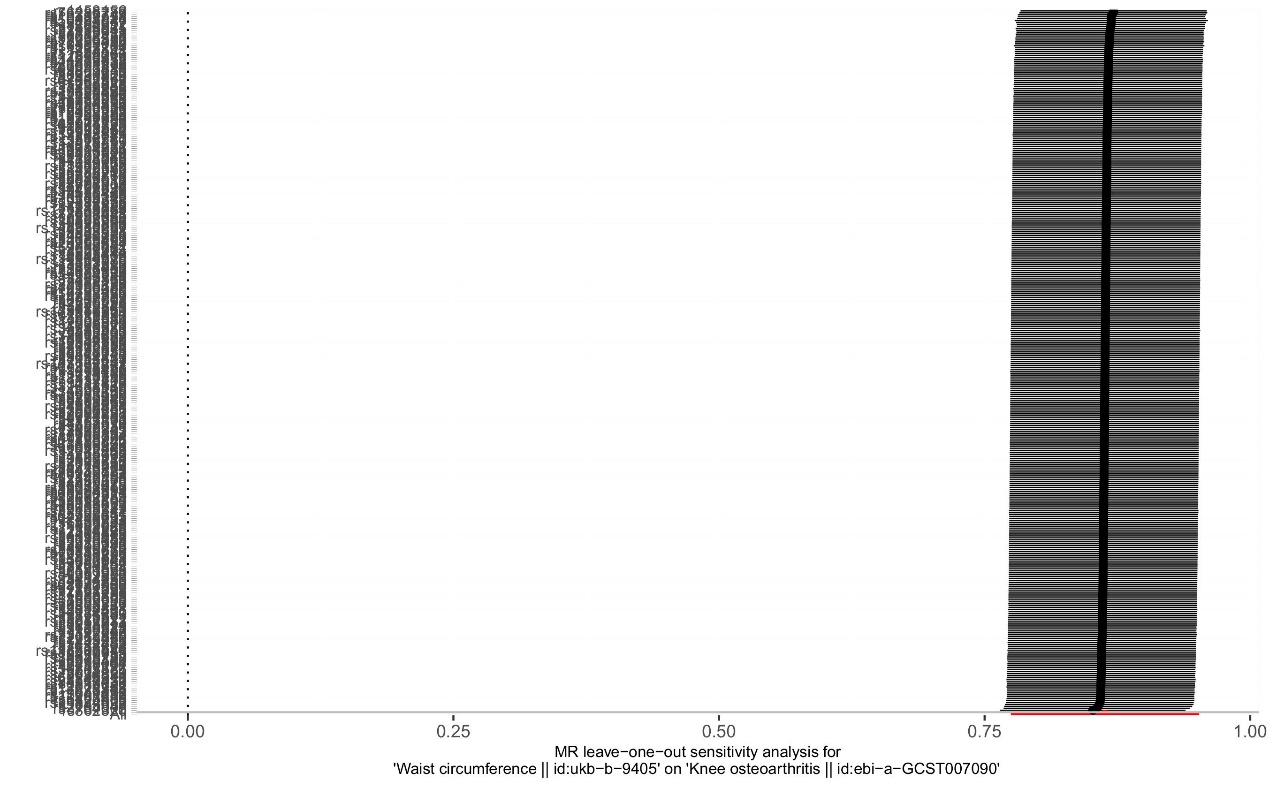
 **Supplementary Figure 24**. A “leave-one-out analysis” plot of waist circumference instruments for knee OA in univariable MR analysis.


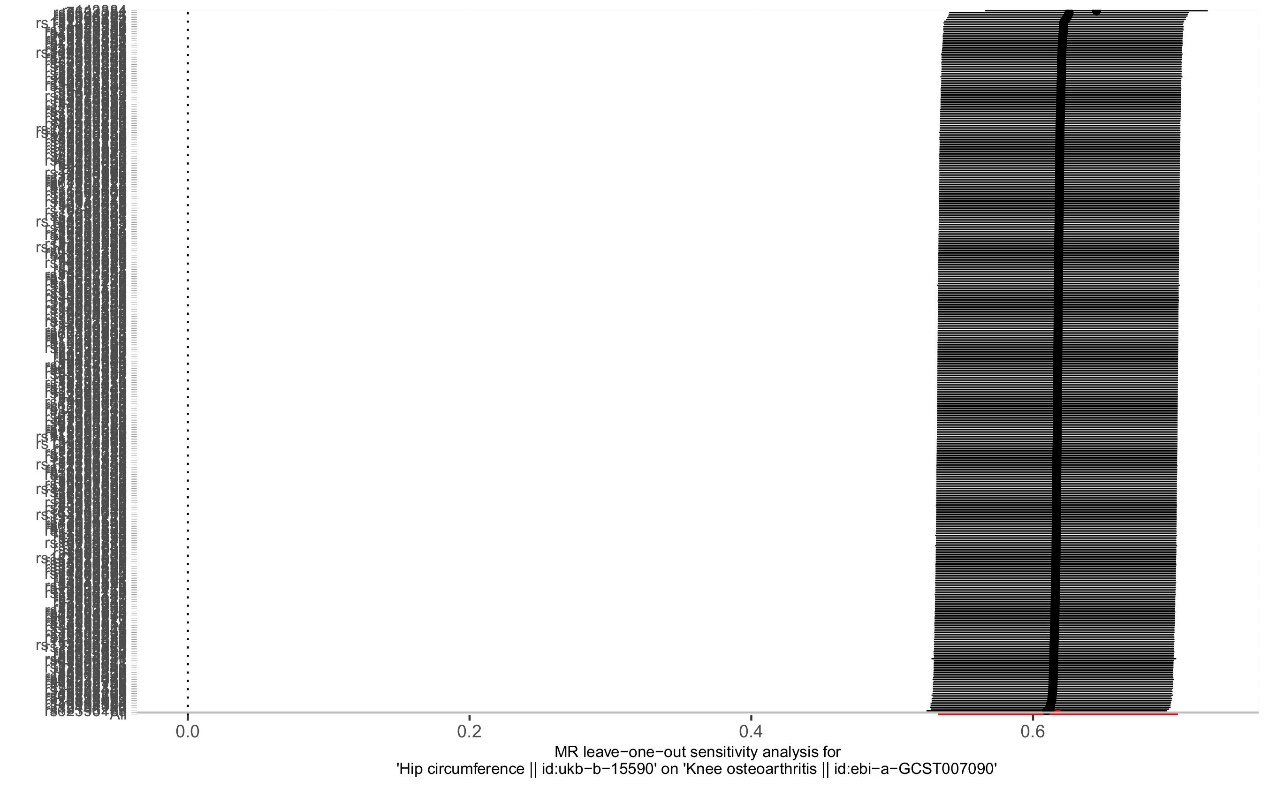
 **Supplementary Figure 25**. A “leave-one-out analysis” plot of hip circumference instruments for knee OA in univariable MR analysis.


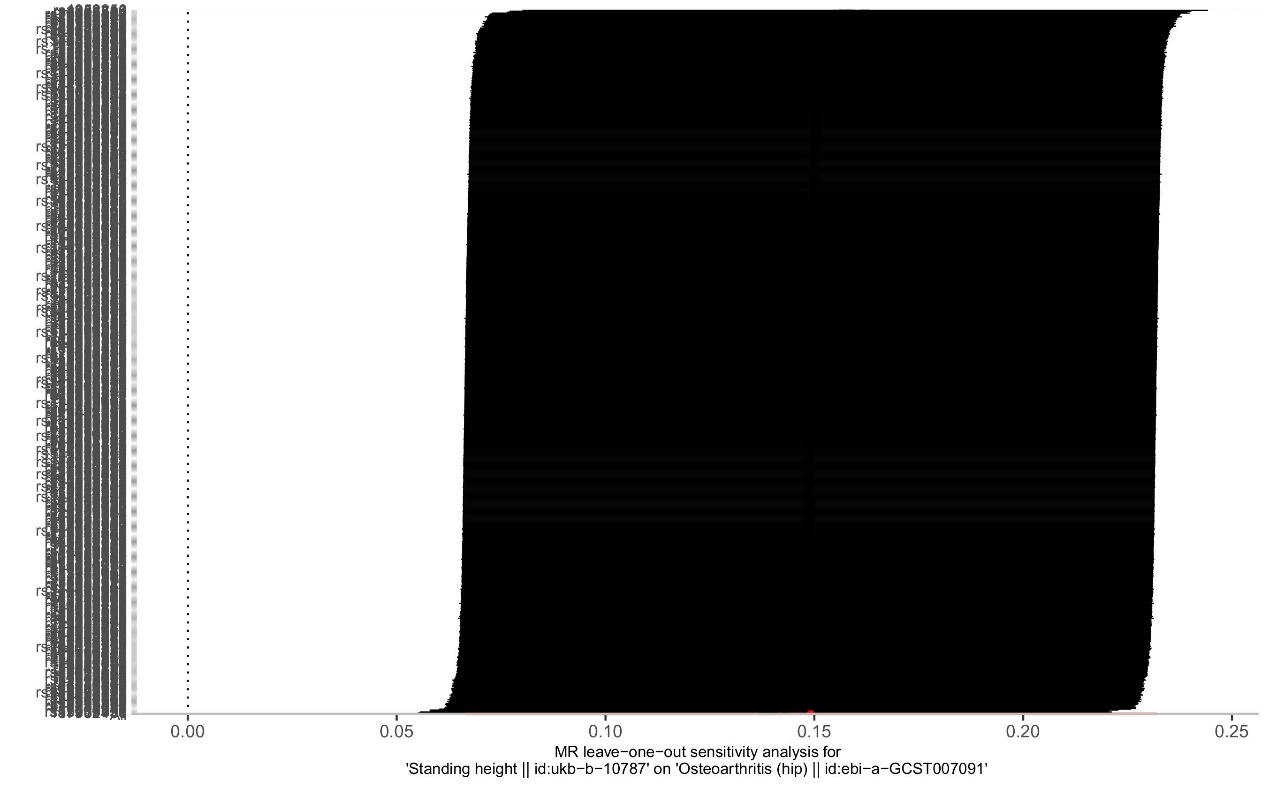
 **Supplementary Figure 26.** A “leave-one-out analysis” plot of height instruments for hip OA in univariable MR analysis.


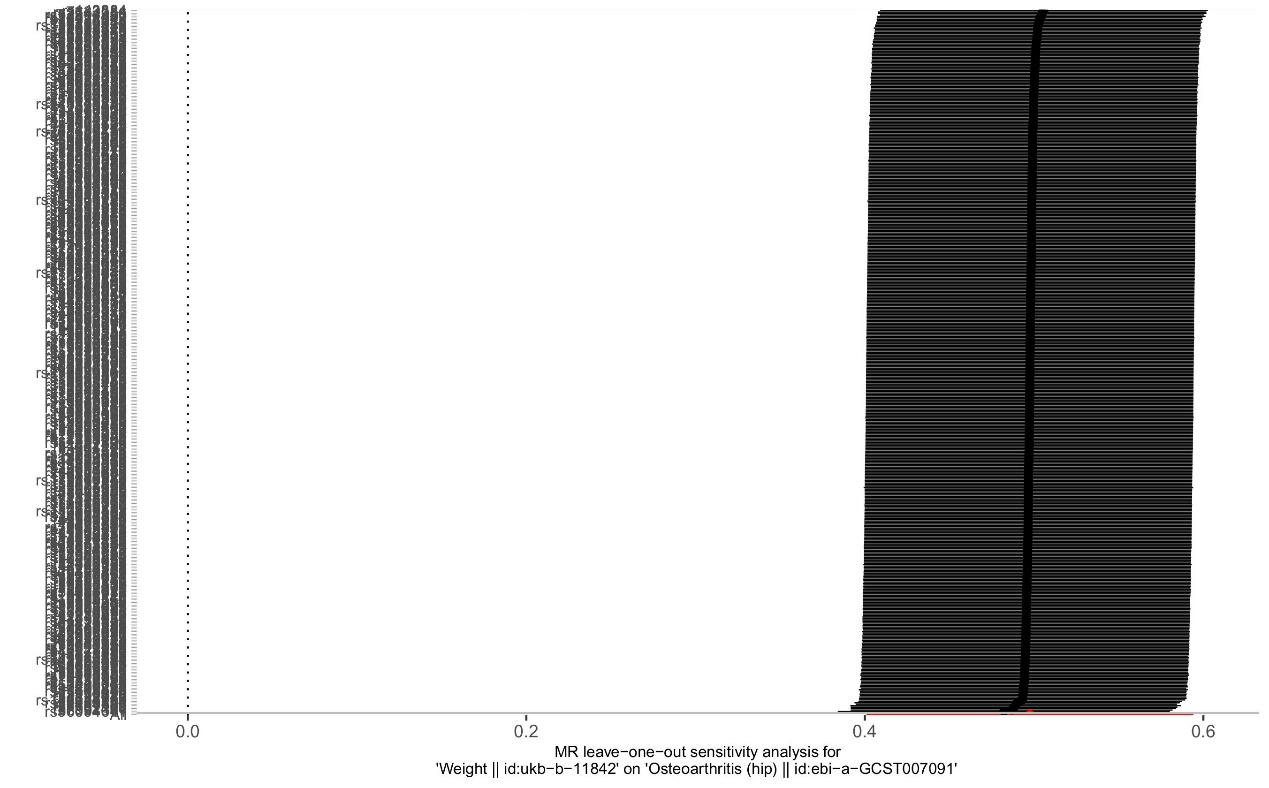
 **Supplementary Figure 27**. A “leave-one-out analysis” plot of weight instruments for hip OA in univariable MR analysis.


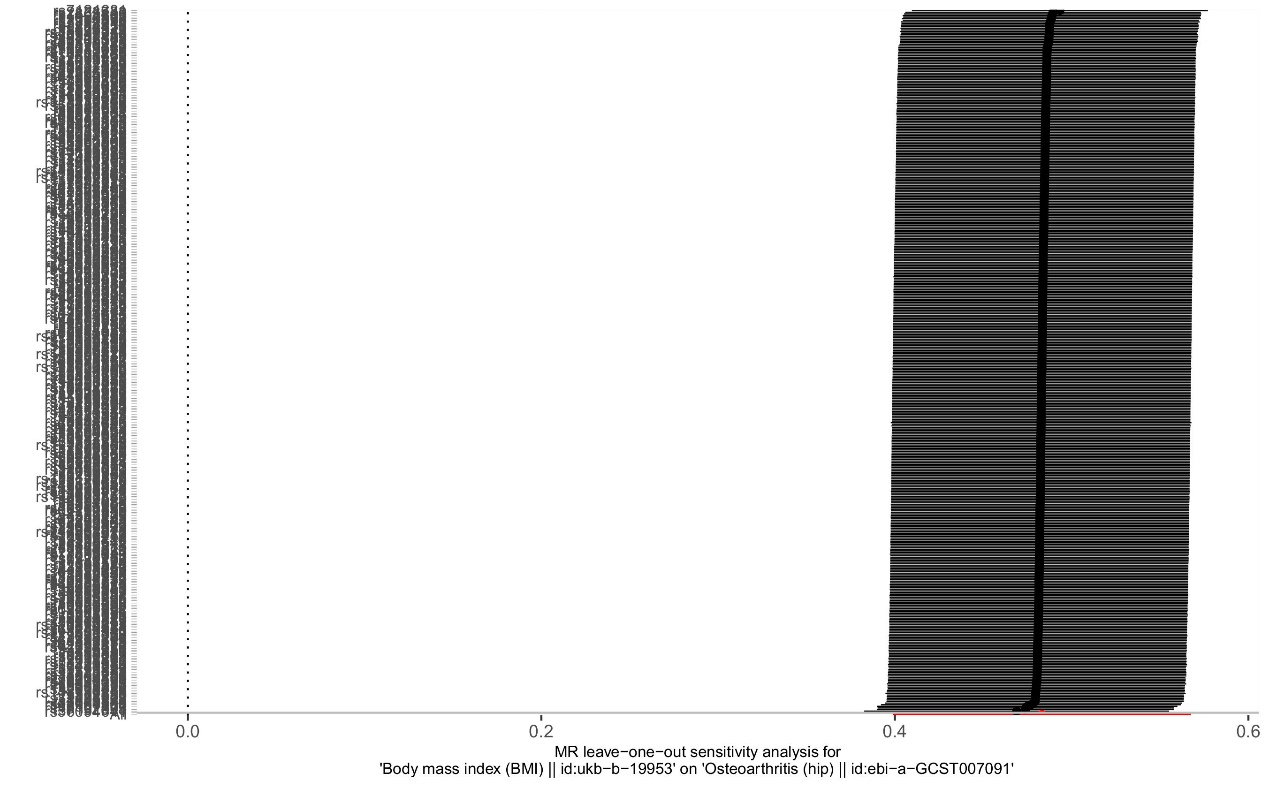
 **Supplementary Figure 28**. A “leave-one-out analysis” plot of BMI instruments for hip OA in univariable MR analysis.


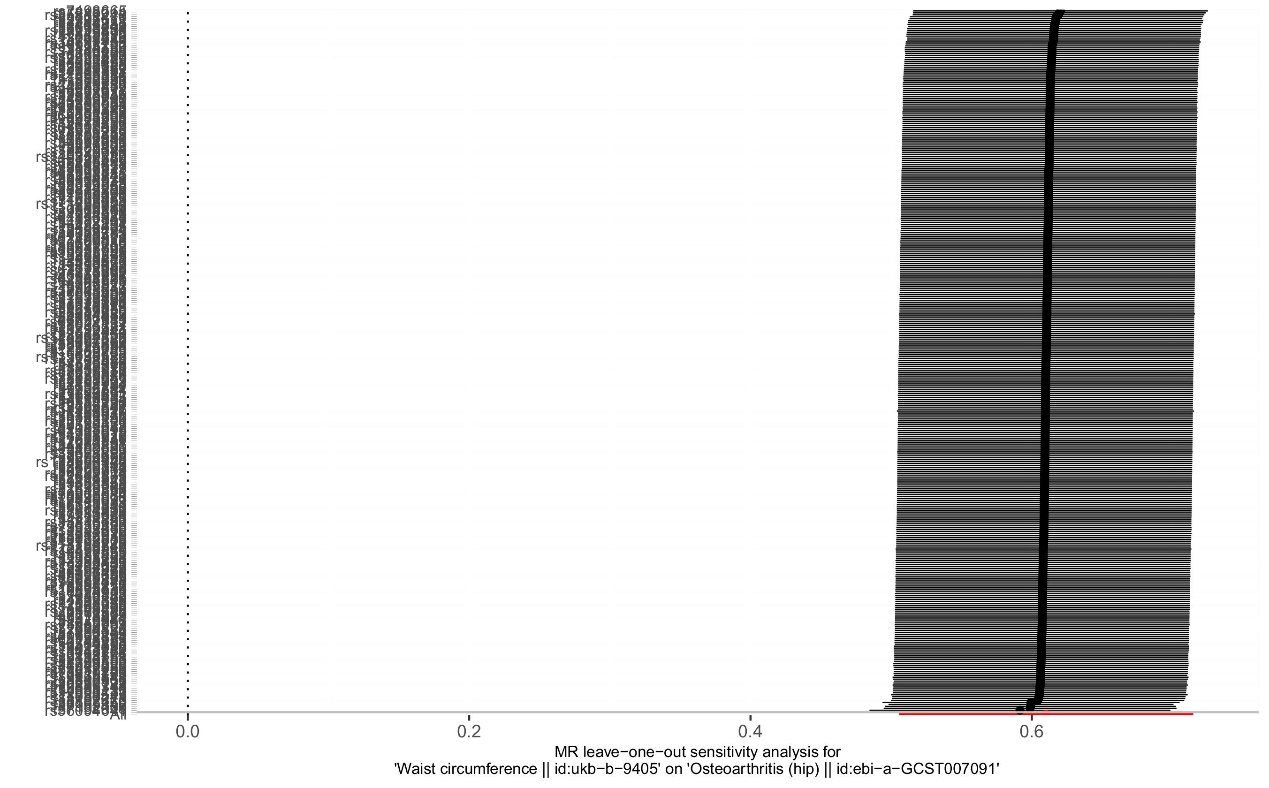
 **Supplementary Figure 29**. A “leave-one-out analysis” plot of waist circumference instruments for hip OA in univariable MR analysis.


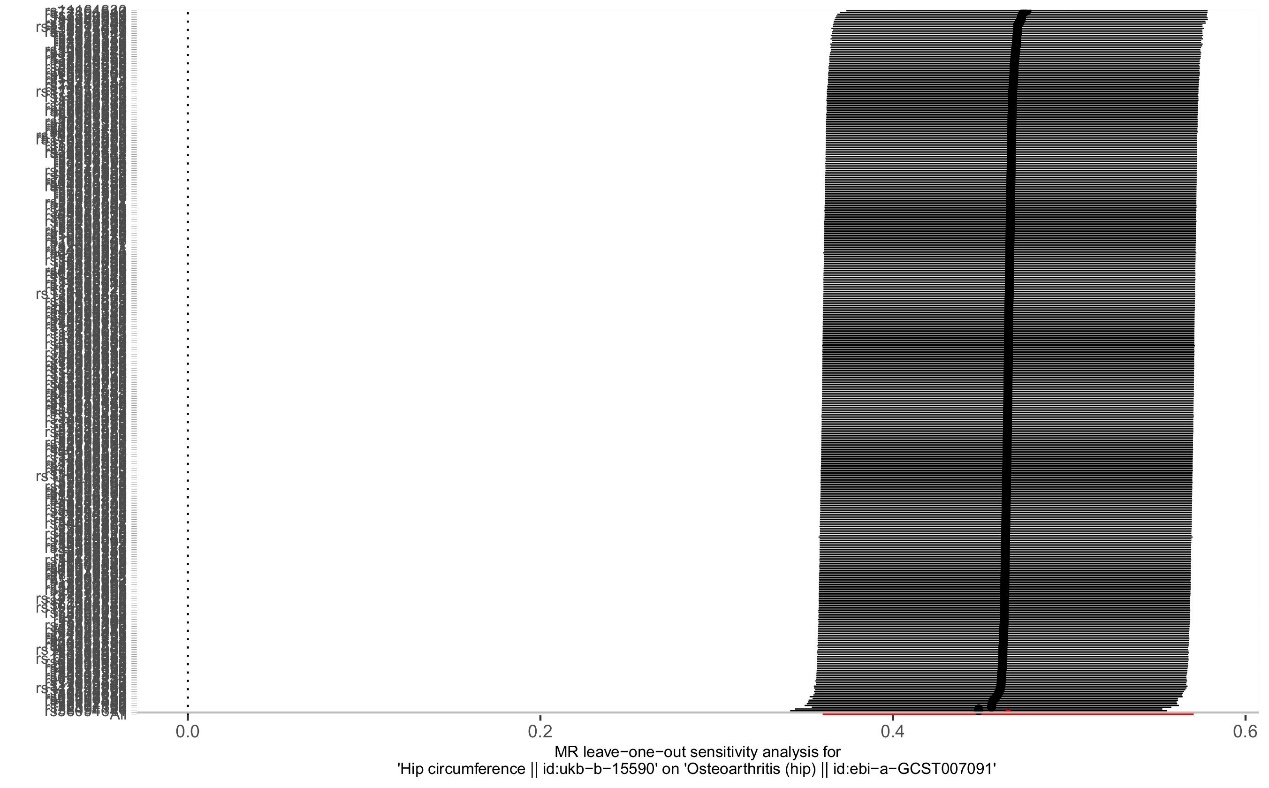
 **Supplementary Figure 30**. A “leave-one-out analysis” plot of hip circumference instruments for hip OA in univariable MR analysis.
